# The ubiquitin-like protein UBTD1 promotes colorectal cancer progression by stabilizing c-Myc to upregulate glycolysis

**DOI:** 10.1038/s41419-024-06890-5

**Published:** 2024-07-13

**Authors:** Liqin Zhao, Nuoya Yu, Yujia Zhai, Yanan Yang, Yixuan Wang, Yue Yang, Zhe Gong, Yanqiu Zhang, Xiaowei Zhang, Weijian Guo

**Affiliations:** 1 Department of Gastrointestinal Medical Oncology, Fudan University Shanghai Cancer Center, Shanghai, China; 2 Department of Oncology, Shanghai Medical College, Fudan University, Shanghai, China; 3 Department of Oncology, Ruijin Hospital, Shanghai Jiao Tong University School of Medicine, Shanghai, China

**Keywords:** Cancer metabolism, Ubiquitylation, Colorectal cancer, Proteolysis

## Abstract

Dysfunction of the ubiquitin-proteasome system (UPS) is involved in the pathogenesis of various malignancies including colorectal cancer (CRC). Ubiquitin domain containing 1 (UBTD1), a ubiquitin-like protein, regulates UPS-mediated protein degradation and tumor progression in some cancer types. However, the biological function and mechanism of UBTD1 are far from being well elucidated, and its role in CRC has not been explored yet. In our study, we analyzed CRC patients’ clinical information and UBTD1 expression data, and found that the expression of UBTD1 in cancer tissue was significantly higher than that in adjacent normal tissue. Higher UBTD1 expression was significantly associated with poorer survival and more lymph node metastasis. Overexpression of UBTD1 could facilitate, while knockdown could inhibit CRC cell proliferation and migration, respectively. RNA-seq and proteomics indicated that c-Myc is an important downstream target of UBTD1. Metabolomics showed the products of the glycolysis pathway were significantly increased in UBTD1 overexpression cells. In vitro, we verified UBTD1 upregulating c-Myc protein and promoting CRC cell proliferation and migration via regulating c-Myc. UBTD1 promoted CRC cells’ glycolysis, evidenced by the increased lactate production and glucose uptake following UBTD1 overexpression. Mechanistically, UBTD1 prolonged the half-life of the c-Myc protein by binding to E3 ligase β-transducin repeat-containing protein (β-TrCP), thereby upregulated the expression of glycolysis rate-limiting enzyme hexokinase II (HK2), and enhanced glycolysis and promoted CRC progression. In conclusion, our study revealed that UBTD1 promotes CRC progression by upregulating glycolysis via the β-TrCP/c-Myc/HK2 pathway, suggesting its potential as a prognostic biomarker and therapeutic target in CRC.

## Introduction

Colorectal cancer (CRC) is one of the most common malignancies worldwide, with over 1.88 million patients diagnosed with CRC and more than 0.91 million dying from it in 2020, making it a major threat to human health globally [1]. However, for advanced or metastatic CRC, the efficacy of traditional treatments, including chemotherapy, radiotherapy, palliative surgery, and target therapies had reached a plateau recently [2, 3]. Though the immune checkpoint inhibitors therapy shows significant anti-tumor activities and become the backbone of the regimes for several cancer types, including microsatellite instability-high CRC, this therapy exhibits marginal benefits for microsatellite stable CRC, which constitutes the majority of this malignancy [4]. Therefore, studies aiming to further clarify the mechanism of CRC progression and identify new targets are critical to improving therapies for advanced or metastatic CRC.

Ubiquitin-proteasome system (UPS) is one of the main approaches to regulate intracellular protein degradation, abnormal regulation of UPS is associated with pathogenesis and development in many types of malignancies [5].

Ubiquitin domain containing 1 (UBTD1), which is a ubiquitin-like protein, could directly interact with the E2 conjugate UBE2D family and likely participate in the UPS cascade in previous reports [6]. UBTD1 has been reported to play significant roles in various cancers, with its downstream mechanisms varying accordingly. In lung, prostate, and hepatocellular cancer, researchers found that UBTD1 is found to catalyze Yes1-associated transcriptional regulator (YAP) protein degradation and inhibit the development of lung, prostate, and hepatocellular cancer [7, 8]. While a study reported recently that UBTD1 may act as a scaffold protein, could promote the binding of substrate proteins such as *N*-acylsphingosine amidohydrolase 1 (ASAH1) and p62 with E3 ubiquitin ligase, thereby facilitating the ubiquitination and degradation of these substrates and regulating the EGFR signaling pathway [9]. Additionally, our previous study found that UBTD1 could promote the degradation of mouse double minute 2 (MDM2) protein, thereby stabilizing p53 protein, leading to cellular senescence in gastric cancer [10]. However, the biological function and mechanism of UBTD1 are still far from fully elucidated, especially in CRC.

This study employed an integrated approach of bioinformatics, experimental analysis, and multi-omics analysis to dissect the role of UBTD1 in CRC. Contrary to previous studies suggesting that UBTD1 suppresses cancer progression, we found that overexpression of UBTD1 could promote CRC cell proliferation and migration, and our analysis revealed that UBTD1 predominantly upregulates glycolysis in CRC cells through a combination of proteomics and metabolomics. Upregulated glycolysis, a well-established marker of tumor metabolic reprogramming [11], is associated with tumor growth, invasion, and drug resistance in CRC [12, 13]. Furthermore, we identified c-Myc protein as the most important downstream target of UBTD1 in CRC cells. c-Myc, as one of the most important oncoproteins, is widely recognized as the essential transcription factor in the glycolysis process in cancer cells [14, 15]. In addition, we revealed that UBTD1 could interact with c-Myc and β-transducin repeat-containing protein (β-TrCP), an E3 ligase, to stabilize the c-Myc protein, therefore upregulate the expression of glycolysis rate-limiting enzyme hexokinase 2 (HK2). Collectively, our study, as detailed below demonstrated that UBTD1 might act as a tumor promoter by upregulating glycolysis via β-TrCP/c-Myc/HK2 pathway in CRC.

## Methods

### Clinical CRC samples

A total of 119 cases of CRC tissues were obtained from the tissue bank of Fudan University Shanghai Cancer Center (FUSCC), and 38 cases of matched adjacent normal tissues were collected simultaneously. Clinical information and survival data of these patients were collected via medical records and follow-up. All patients had pathologically confirmed colorectal adenocarcinoma, and samples from these patients were kept in RNAlater at −80 °C immediately after the tumor being removed. Total RNA was extracted from these samples for Quantitative real-time polymerase chain reaction (qRT-PCR) analysis. The collection and use of these tissues were approved by the Institutional Medical Ethics Committee of the FUSCC, informed consent was obtained from all patients, and the study protocol was in accordance with the ethical guidelines of the Declaration of Helsinki. The clinical information and gene expression data of TCGA CRC samples (*n* = 589) were downloaded from the FireBrowse database (http://www.firebrowse.org/).

### Cell culture

Four CRC cell lines and a normal colon epithelial cell line were used in this study, SW620, HCT116, P53R, RKO, and NCM460 were purchased from Shanghai Cell Bank of Type Culture Collection, Chinese Academy of Sciences (Shanghai, China). HEK 293FT cell line was obtained from the American Type Culture Collection (ATCC). All cell lines were authenticated via short tandem repeat (STR) profiling. Cells were cultured in high-glucose DMEM medium supplemented with 10% fetal bovine serum (FBS) and 1% penicillin-streptomycin solution, and cells were passaged every 2–4 days according to cell density.

### Plasmids, siRNAs, and stable cell line establishment

The lentiviral vectors expressing UBTD1, UBTD1 shRNA, c-Myc, and matched empty vectors were obtained from Hanyin Biotechnology Limited Company (Shanghai, China). The UBTD1 overexpression plasmid was constructed by cloning the full-length of UBTD1 cDNA into CMV-MCS-PGK-Puro vector, and c-Myc overexpression plasmid was constructed by cloning the full-length of c-Myc cDNA into CMV-MCS-PGK-Blasticidin vector. Plasmids carrying shRNA targeting UBTD1 were generated using the hU6-MCS-CMV-ZsGreen1-PGK-Puro vector. The targeted sequences of UBTD1 were 5′-GCTTAAGTGGAAGAGCGAC-3′ and 5′-TCATGGCACCCTCTGTGAA-3′. The plasmids carrying shRNA targeting c-Myc, HK2, and β-TrCP were obtained from GenePhama (Shanghai, China), and the targeted sequences were 5′-GAACATCATCATCCAGGAC-3′, 5′-GCCTGGCTAACTTCATGGATA-3′, and 5′-GCCTGGCTAACTTCATGGATA-3′ respectively. The inserted fragments were confirmed by Sanger sequencing. UBTD1 overexpression or knockdown stable cell lines were generated through infecting colorectal cells with respective lentiviruses and sorted by antibiotics or flow cytometry. Overexpression or knockdown of UBTD1 was determined at both mRNA and protein levels by qRT-PCR and western blot.

### RNA extraction and qRT-PCR

Total RNA was extracted from CRC cells or CRC samples using TRIzol reagent (Invitrogen, CA, USA). mRNA reverse transcription was performed using PrimeScript^TM^ RT reagent Kit with gDNA Eraser (RR047A, Takara, Japan). qRT-PCR was conducted using SYBR Premix Ex Taq^TM^ II (RR820A, Takara, Japan) according to the manufacturer’s protocols in an ABI 7900 Real-time PCR system (Life Technologies, USA). The primers for GAPDH, UBTD1, c-Myc, HK2, FBXW1A, and FBXW1B are listed in Supplementary Table 1.

### Western blot and antibodies

RIPA lysis buffer supplemented with protease inhibitors was used to extract the whole cell lysates. The protein concentration of each sample was determined by the Bicinchoninic Acid Assay. Western blotting was performed as described in our previous studies [16]. The antibodies used in this study are as follows: anti-UBTD1 (HPA034825, Sigma-Aldrich, MO, USA), anti-c-Myc (13987, Cell Signaling Technology, MA, USA), anti-β-TrCP (4394, Cell Signaling Technology, MA, USA), anti-β-actin (66009-1-Ig, Proteintech, IL, USA), anti-GAPDH (60004-1-Ig, Proteintech, IL, USA), anti-HK1 (19662-1-AP, Proteintech, IL, USA), anti-HK2 (22029-1-AP, Proteintech, IL, USA), anti-LDHA (19987-1-AP, Proteintech, IL, USA), anti-LDHB (14824-1-AP, Proteintech, IL, USA), anti-GLUT3 (20403-1-AP, Proteintech, IL, USA), and anti-ALDOA (11217-1-AP, Proteintech, IL, USA).

### Cell proliferation, colony formation, and cell migration assay

Cell Counting Kit-8 (CK04, Dojindo, Japan) was used to assess CRC cell proliferation. Briefly, 4 × 10^3^ cells were seeded in each well with three replicates in 96-well plates. Cell numbers were determined on day 1, day 2, day 3, day 4, and day 5 using CCK-8. For colony formation assay, 1–2 × 10^3^ cells were seeded in each well with three replicates in six-well plates, after cultured for 2 weeks, culture medium was removed and colonies were fixed with 4% paraformaldehyde for 20 min and stained with 0.5% crystal violet for 10 min. Finally, colonies were washed, photographed, and counted. Transwell chamber assay was applied to detect cell migration. Briefly, cells were harvested and counted using an automatic cell counter (Beckman Coulter, USA), then resuspended in a serum-free medium to achieve a concentration of 4 × 10^5^ cells/ml. 200 μL of cell suspension, which contains 8 × 10^4^ cells was added into the upper chambers which were placed in 24-well plates, while 600 μL of medium supplemented with 20% FBS was added in the lower chamber. Twenty-four to forty-eight hours later, migrated cells which were on the lower side of the membrane were fixed, stained, and counted.

### RNA-seq

The total RNA of UBTD1 overexpressing HCT116 cells and control cells were extracted using TRIzol reagent, the RNA concentration was determined with NanoDrop spectrophotometer (Life Technologies, USA). The quality and integrity of RNA were evaluated by agarose gel electrophoresis. RNA-seq was performed by Majorbio Technology Co. Ltd (Shanghai, China) using the Illumina HiseqTM2500. Firstly, mRNA was purified from total RNA using poly-T oligo-attached magnetic beads, and randomly cleaved into small fragments using RNA fragmentation buffer. cDNA library was established with randomly cleaved mRNA as a template, and a quality inspection of the cDNA library was conducted. Then cDNA library was sequenced on the Illumina HiseqTM2500 platform. The process of sequencing data includes raw data acquisition, raw data infiltration, reference sequence comparison, and analysis.

### Proteomics

UBTD1 overexpression and control HCT116 cells were expanded and collected. Proteomics were performed by Applied Protein Technology Co., Ltd (Shanghai, China) using a liquid chromatography– tandem mass spectrometry (LS-MS/MS) system with the Tandem Mass Tags labeling method. The control group and UBTD1 overexpression group were repeated for three times, and about 1 × 10^7^ cells were required for each repetition.

### Metabolomics

UBTD1 overexpression and control HCT116 cells were expanded, collected, and sent to Metabo-Profile Biotechnology Co., Ltd. (Shanghai, China) for Q300^TM^ metabolomics detection using an ultra-performance liquid chromatography coupled to tandem mass spectrometry (UPLC-MS/MS) system. The control group and UBTD1 overexpression group were repeated six times, and each repetition needed 1 × 10^7^ cells.

### Lactate production and glucose uptake detection

Lactate Colorimetric Assay Kit II (K627-100, BioVision, SF, USA) was used to detect the lactate level in the culture medium. Briefly, 8 × 10^4^ cells were inoculated per well in a 12-well plate, the culture medium was replaced with 500 μL of serum-free medium after the cells adhered to the wall. Half an hour later, the culture medium was collected, and lactate concentration was measured by the above lactate assay kit according to the operation instructions. Glucose Uptake Colorimetric Assay Kit (K676-100, Biovision, SF, USA) and Glucose (GO) Assay Kit (GAGO20, Sigma-Aldrich, MO, USA) were used to measure cell glucose uptake according to the manufacturer’s protocol.

### Analysis of protein stability

Cycloheximide chase assay was applied to assess protein stability. Briefly, Cells were seeded in six-well plates with a density that can reach 90% fusion the next day. Twenty-four hours later, cells were treated with 100 μg/ml cycloheximide (HY-12320, MCE, NJ, USA) for the indicated time. Total cell protein was harvested, protein concentration was determined by Bicinchoninic Acid Assay and adjusted to the same concentration, and the level of c-Myc and β-actin protein was detected by western blot. The c-Myc protein levels were calibrated to corresponding β-actin protein levels using Image J.

### Co-immunoprecipitation (co-IP)

Dynabeads^TM^ protein G (10003D, Invitrogen, CA, USA) was applied to carry out protein co-immunoprecipitation. Briefly, cells were cultured in 15 cm culture dishes, then we discarded the culture medium and washed the cells with precooled PBS three times. Cells were lysed with moderate western and IP cell lysate on ice for 1 h. Meanwhile, 50 μl dynabeads^TM^ protein G was incubated with 3 μg antibody of target protein or IgG for 1 h at room temperature. Centrifuge the cell lysate and collect supernatant, then mix the supernatant with beads-antibody complex, and incubate overnight at 4 °C. Wash the complex of beads-antibody-bound proteins with lysis buffer for three times, and detect bound proteins and inputs by immunoblotting.

### In vivo ubiquitination assay

HEK 293FT cells were transfected with different combinations of plasmids encoding UBTD1, c-Myc, and His-Ub. Cell proteins were harvested 48 h after transfection, and cells were treated with 20 μM MG132 (HY-13259, MCE, NJ, USA) for 6 h before protein harvest. c-Myc protein was immunoprecipitated with c-Myc antibody. About 80 μl cell lysis of each sample was left as positive control and internal reference before immunoprecipitation. The ubiquitination of c-Myc protein was detected with ubiquitin antibody by WB.

### Subcutaneous xenograft model

Animal studies were approved by the Institutional Animal Care and Use Committee of Fudan University, with each experiment involving three or four groups of mice. For statistical comparisons, we utilized the mean values of each group as parameters for assessing inter-group differences. The number of mice in each group should be equal to twice the number of groups, resulting in approximately six mice per group. 5- to 6-weeks old male BALB/c nude mice with average 20 g body-weight were obtained from Shanghai SLAC Laboratory Animal Co., Ltd and raised under specific pathogen-free (SPF) condition, all the mice were assigned to each group without randomization due to the low individual- heterogeneity. About 5 × 10^6^ UBTD1 overexpressing and control HCT116 cells suspended in 200 μl sterile PBS were injected into the flanks of randomly grouped mice (*n* = 10 for each group). Observe every 2 or 3 days, after the appearance of tumors, five mice were randomly selected from each group and treated with 2-DG by intraperitoneal administration at the dose of 500 mg/kg. The total dose was calculated according to the weight of the mice. After the first administration, 2-DG was administered every 4 days, with a total of three intraperitoneal injections. Four weeks after inoculation, all mice were sacrificed, the subcutaneous tumors were harvested, and the size and weight of tumors were measured and weighed.

### Pulmonary metastasis model by tail vein injection

Nude mice were injected with 2.5 × 10^6^ UBTD1 overexpressing or control HCT116 cells suspended in 200 μl sterile PBS via tail vein (*n* = 10 per group), no blinding was implemented in this experiment. On the third day after injection, five mice were randomly selected from each group and treated with 2-DG through intraperitoneal administration at the dose of 500 mg/kg. Similarly, 2-DG was administered every four days with a total of three intraperitoneal injections. Eight weeks later, all mice were sacrificed and lung metastatic loci were examined by hematoxylin-eosin staining under a microscope. Five random slices from each mouse lung were used for the calculation of metastatic sites.

### Statistics

All statistical analysis was performed using R language (version 3.5.1). In the current manuscript, we performed a post-hoc analysis utilizing data from our hospital and publicly accessible data from TCGA. All statistical analyses conducted were descriptive in nature, without the formulation of statistical hypotheses. The clinical Two-tailed student’s *t*-test or one-way ANOVA were applied to compare the difference between or among different groups if not stated specifically. Survival analysis was performed using the Kaplan–Meier method and analyzed by log-rank test and Cox proportional risk model. Pearson chi-square test was used to examine the correlation between UBTD1 expression and clinicopathologic characteristics. Univariate and multivariate regression analysis were used to determine prognosis risk factors. The difference is considered statistically significant if the *P* value is less than 0.05. The in vitro experiments in our study were repeated three times, and the data was described as mean ± SD.

## Results

### Upregulation of UBTD1 is associated with unfavorable prognosis in patients with colorectal cancer

**Fig. 1:**
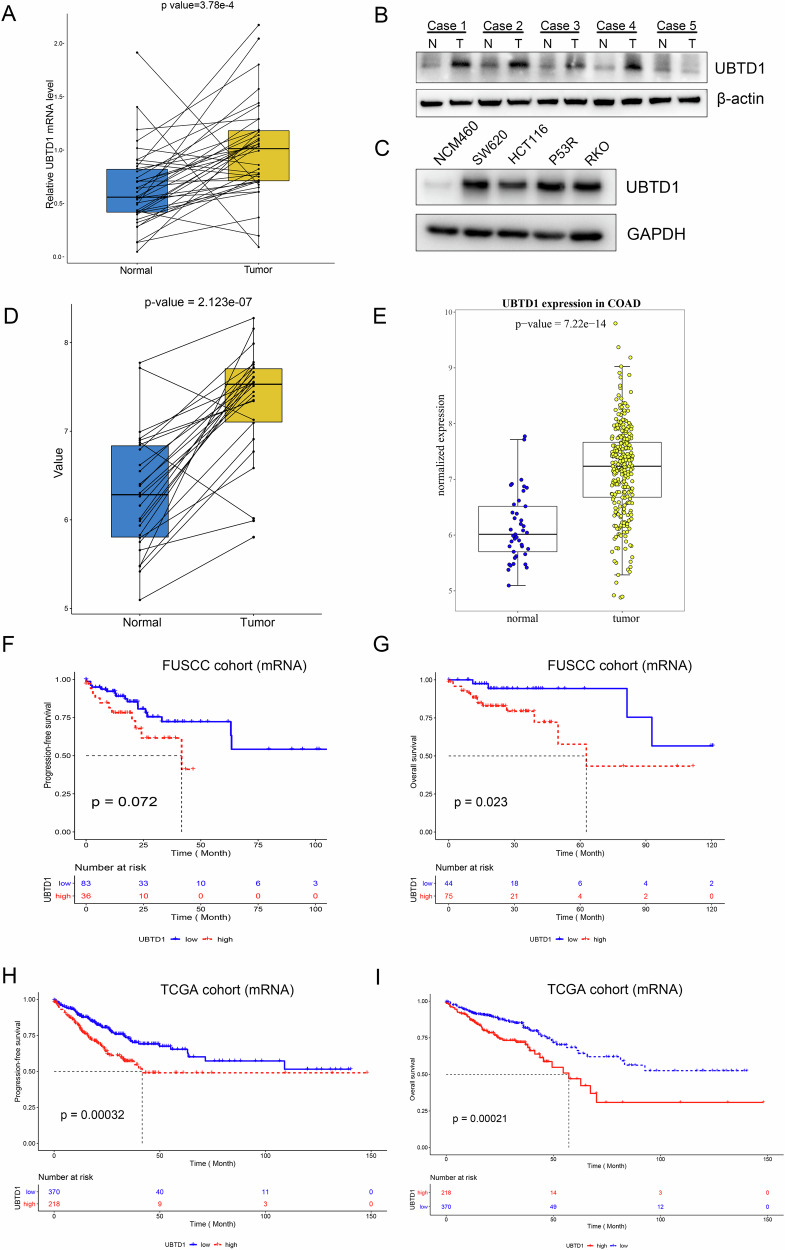
UBTD1 is upregulated in colorectal cancer tissues and cell lines, upregulation of UBTD1 is associated with unfavorable prognosis in colorectal cancer patients. **A** The expression of UBTD1 mRNA in 38 paired tumor tissues and adjacent normal tissues from FUSCC CRC patients was analyzed with a paired *t*-test. **B** The expression of UBTD1 protein in five paired tumor tissues and adjacent normal tissues from FUSCC CRC patients. **C** The protein expression of UBTD1 in NCM460 (derived from colon epithelial cells) and colorectal cancer cell lines including SW620, HCT116, P53R, and RKO. **D** The expression of UBTD1 mRNA in paired tumor tissues and adjacent normal tissues from TCGA CRC patients was analyzed with a paired *t*-test. **E** The expression of UBTD1 mRNA in tumor tissues and normal tissues from TCGA CRC patients was analyzed with a *t*-test. **F** The Kaplan–Meier curve of progression-free survival in UBTD1-high expression patients versus UBTD1-low expression patients from the FUSCC cohort. **G** The Kaplan–Meier curve of overall survival in UBTD1-high expression patients versus UBTD1-low expression patients from the FUSCC cohort. **H** The Kaplan–Meier curve of progression-free survival in UBTD1-high expression patients versus UBTD1-low expression patients from TCGA cohort. **I** The Kaplan–Meier curve of overall survival in UBTD1-high expression patients versus UBTD1-low expression patients from the TCGA cohort.

In order to understand the potential biological function of UBTD1 in colorectal cancer, we first examined the expression of UBTD1 in CRC samples and adjacent normal tissues, and its association with patients’ survival. Compared to adjacent normal tissues, the expression of UBTD1 mRNA in colorectal cancer was significantly higher in 38 pairs of colorectal cancer and corresponding adjacent normal tissues from FUSCC (*P* < 0.001, Fig. 1A). In addition, total protein was extracted from five pairs of samples, and the expression level of UBTD1 protein was detected by WB. It was found that UBTD1 was upregulated in cancer tissues in most cases (four cases/five cases) (Fig. 1B). Meanwhile, we examined the expression of UBTD1 protein in a normal colon epithelial cell line NCM460 and four CRC cell lines SW620, HCT116, P53R, and RKO, and found higher expression in CRC lines (Fig. 1C). Consistent with the findings in our own dataset, the expression of UBTD1 was higher in CRC tissues than that in adjacent normal tissues from The Cancer Genome Atlas (TCGA) dataset both in paired samples (Fig. 1D) and in grouped samples (Fig. 1E). We explored the association between UBTD1 expression and clinicopathological characteristics of CRC patients using data from TCGA, and found that UBTD1 expression was associated with lymph node metastasis and TNM stage, but not significantly correlated with age, gender and cancer types (Supplementary Table 2). The association between UBTD1 expression and clinical features within the FUSCC datasets was further scrutinized. The analysis revealed that CRC patients with stage II and stage III disease exhibited elevated levels of UBTD1 expression, however, these differences did not reach statistical significance (Supplementary Table 3). Gene set enrichment analysis (GSEA) based on TCGA CRC datasets suggested that epithelial mesenchymal transition (EMT), hypoxia, and inflammatory response pathways were significantly enriched in UBTD1-high CRC cases (Supplementary Fig. 1A). UBTD1 mRNA level is positively correlated with multiple EMT related markers, including CDH2, vimentin, MMP9, Snail, Slug, but negatively correlated with epithelial marker CDH1 (Supplementary Fig. 1B). Survival analysis revealed that higher level of UBTD1 is significantly correlated with unfavorable progression-free survival and overall survival both in FUSCC cohort and TCGA cohort (Fig. 1F–I). Additionally, univariate and multivariate analysis of the association between UBTD1 expression and overall survival showed that UBTD1 was an independent prognostic factor for CRC patients (Supplementary Table 4). Taken together, high UBTD1 expression may represent an unfavorable prognostic for CRC patients.

### UBTD1 promotes cell proliferation and migration in CRC cells

**Fig. 2:**
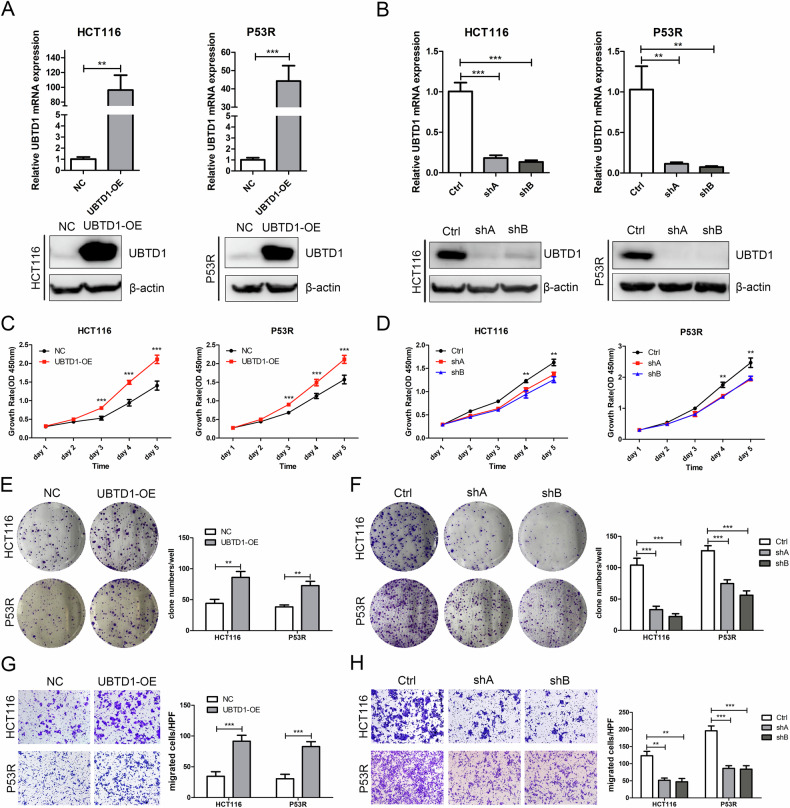
UBTD1 promotes the proliferation and migration of CRC cells. **A** The UBTD1 mRNA and protein were detected using qRT-PCR and WB to validate the overexpression of UBTD1 in HCT116 and P53R cell lines. **B** The UBTD1 mRNA and protein were detected using RT-PCR and WB to validate the knockdown of UBTD1 in HCT116 and P53R cell lines. **C** CCK-8 was used to assess the proliferation after UBTD1 overexpression in HCT116 and P53R cell lines. **D** CCK-8 was used to assess the proliferation after UBTD1 knockdown in HCT116 and P53R cell lines. **E** Colony formation assay was used to assess the proliferation after UBTD1 overexpression in HCT116 and P53R cell lines. **F** Colony formation assay was used to assess the proliferation after UBTD1 knockdown in HCT116 and P53R cell lines. **G** Transwell chamber assay was used to assess migration after UBTD1 overexpression in HCT116 and P53R cell lines; **H** Transwell chamber assay was used to assess migration after UBTD1 knockdown in HCT116 and P53R cell lines. **P < 0.01 or *** *P* < 0.001 indicates a significant difference between the indicated groups.

To further identify the biological function of UBTD1 in CRC, we examined the subcellular localization of UBTD1 in CRC cells and found that UBTD1 is mainly located in the cytoplasm rather than the nucleus (Supplementary Fig. 1C). UBTD1 overexpression and knockdown stable cell lines were constructed by transfecting CRC cells with lentiviruses. The overexpression or knockdown of UBTD1 mRNA and protein in HCT116 and P53R stable cell lines was verified by qRT-PCR and WB (Fig. 2A, B). CCK-8 and colony formation assay were applied to assess cell proliferation after UBTD1 overexpression or knockdown. We found UBTD1 overexpression promoted CRC cell proliferation (Fig. 2C), while UBTD1 knockdown inhibited the proliferation of CRC cells (Fig. 2D). Overexpression of UBTD1 increased clony formation (Fig. 2E), whereas UBTD1 knockdown exhibited opposite effect (Fig. 2F). Transwell migration assay showed UBTD1 overexpression significantly facilitated CRC cell migration (Fig. 2G), while UBTD1 knockdown suppressed ability of migration (Fig. 2H). Taking together, the above in vitro experiments demonstrated that UBTD1 plays a tumor-promoting role in CRC.

### UBTD1 promotes CRC proliferation and migration by regulating c-Myc protein level depending on its ubiquitin domain

**Fig. 3:**
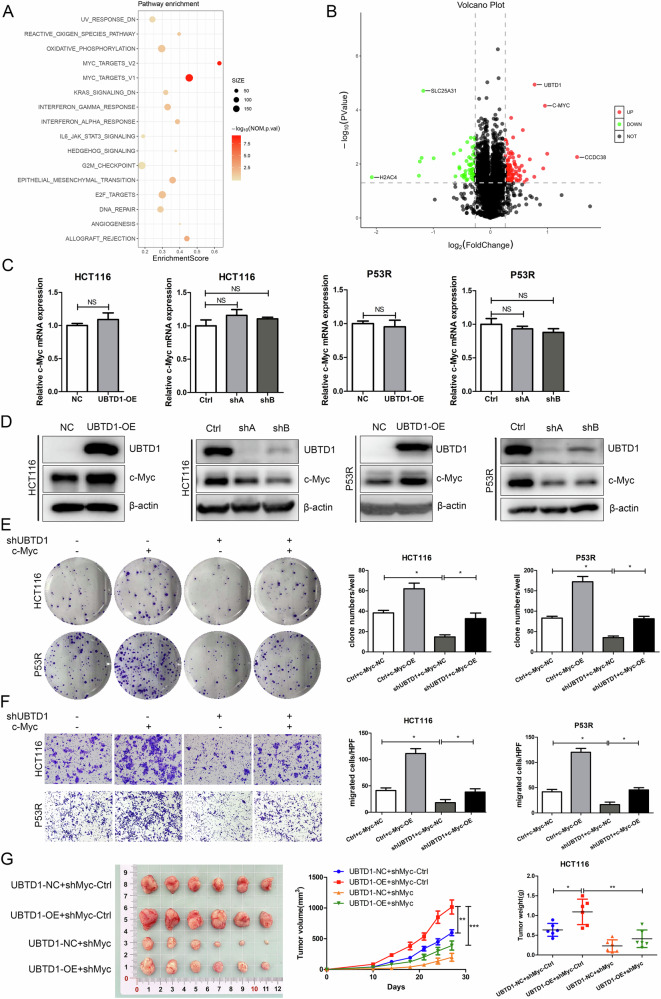
UBTD1 promotes colorectal cancer cell proliferation and migration by regulating c-Myc protein. **A** Gene set enrichment analysis based on the RNA-seq in UBTD1 overexpression HCT116 cells compared to the control HCT116 cells. **B** Volcano plot based on proteomics in UBTD1 overexpression HCT116 cells compared to control HCT116 cells. **C** The mRNA expression of c-Myc in UBTD1 overexpression or knockdown HCT116 and P53R cells. **D** The protein expression of c-Myc in UBTD1 overexpression or knockdown HCT116 and P53R cells. **E** Decreased colony formation caused by UBTD1 knockdown can be rescued by upregulation of c-Myc in HCT116 and P53R cells. **F** Decreased migration capability caused by UBTD1 knockdown can be rescued by upregulation of c-Myc in HCT116 and P53R cells. **G** Downregulation of c-Myc expression effectively reverses the augmented xenograft tumor proliferation facilitated by overexpression of UBTD1. *P < 0.05, **P < 0.01, or *** *P* < 0.001 indicates a significant difference between the indicated groups.

To clarify the underlying mechanism of UBTD1 in regulating the proliferation and metastasis of CRC cells, we performed RNA-seq and proteomics in UBTD1-overexpression and control HCT116 cells. Gene Set Enrichment Analysis (GSEA) based on RNA-seq indicated that MYC signaling was greatly enriched in UBTD1-overexpressing cells (Fig. 3A and Supplementary Fig. 1D). Proteomics data showed that the level of c-Myc protein in UBTD1-overexpressing cells increased significantly compared to control cells (Fig. 3B). However, previously reported UBTD1 target proteins were not affected by UBTD1 overexpression in proteomics, such as TP53 and YAP. Protein–protein interaction (PPI) analysis based on proteomics suggested that c-Myc (connectivity = 7) may be the central node of protein interaction (Supplementary Fig. 2A), which indicated c-Myc might be an important downstream target of UBTD1. In order to further clarify whether UBTD1 regulates the expression of c-Myc, the level of c-Myc mRNA and protein was determined in UBTD1 overexpression and knockdown cells. We found that UBTD1 had no effect on the level of c-Myc mRNA (Fig. 3C), but can regulate c-Myc protein level. UBTD1 overexpression significantly increased c-Myc protein, whereas knockdown of UBTD1 dramatically downregulated c-Myc protein (Fig. 3D). To investigate whether UBTD1 regulates c-Myc protein depending on its ubiquitin domain, we investigated the effect of mutated-UBTD1 that removed ubiquitin domain (Mut) on c-Myc protein, which had been demonstrated to be loss of function in our previous study [10]. We discovered that overexpression of mutated-UBTD1 had an effect on the c-Myc protein (Supplementary Fig. 2B) and cell proliferation and migration (Supplementary Fig. 2C, D), which indicated the ubiquitin domain was essential for UBTD1 to perform its biological function. In order to clarify whether UBTD1 promotes CRC progression via regulating c-Myc, we overexpressed c-Myc in UBTD1 knockdown cells to determine whether c-Myc overexpression could restore the malignant phenotype inhibited by UBTD1 shRNA. The results showed that overexpression of c-Myc could reverse the inhibitory effect of UBTD1 interference on CRC cell colony formation and migration (Fig. 3E, F). Additionally, in vivo studies have successfully demonstrated that the knockdown of c-Myc expression effectively reverses the augmented xenograft tumor proliferation facilitated by overexpression of UBTD1 (Fig. 3G). Collectively, the above results demonstrated that UBTD1 regulated CRC malignant phenotype via c-Myc.

### UBTD1 promotes aerobic glycolysis by regulating the expression of HK2, the first rate-limiting enzyme of glycolysis, via c-Myc

**Fig. 4:**
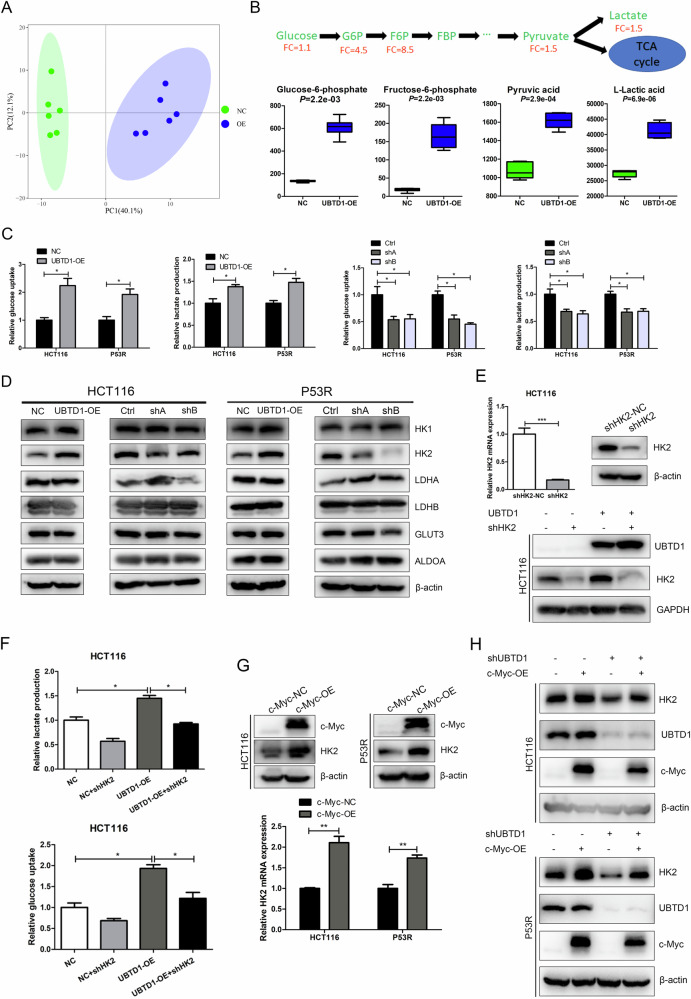
UBTD1 promotes aerobic glycolysis in colorectal cancer cells by regulating the expression of hexokinase 2 via upregulating c-Myc. **A** Principal component analysis showed a distinctive principal component 1 pattern between the UBTD1 overexpression group and the control group. **B** Enhanced glycolysis was observed in UBTD1 overexpression HCT116 cells with elevated G6P, F6P, pyruvate, and lactate via metabonomics analysis. **C** The overexpression and knockdown of UBTD1 can respectively increase or decrease glucose uptake and lactate production in HCT116 and P53R cells. **D** Several key glycolysis-related genes were detected by WB in UBTD1 overexpression or knockdown HCT116 and P53R cells, showing HK2 was significantly associated with UBTD1 expression. **E** The construction of HK2 interfering plasmid, and validation in UBTD1 overexpressed or control HCT116 cells. **F** The promotion effect on lactate production and glucose uptake after UBTD1 overexpression could be diminished by HK2 knockdown in HCT116 cells. **G** HK2 was significantly upregulated after c-Myc overexpression both at mRNA and protein levels through qRT-PCR and WB assay in HCT116 and P53R cells. **H** Downregulation of HK2 caused by UBTD1 knockdown can be rescued by overexpression of c-Myc in HCT116 and P53R cells. *P < 0.05, **P < 0.01, or ***P < 0.001 indicates a significant difference between the indicated groups.

Gene Ontology (GO) based on proteomics revealed that UBTD1 participated in metabolic processes and biological regulation (Supplementary Fig. 3A). In addition, KEGG pathway analysis indicated that UBTD1 may be involved in signal pathways related to glucose metabolism, including oxidative phosphorylation, glycolysis/ gluconeogenesis, fructose and mannose metabolism, etc. (Supplementary Fig. 3B). Energy metabolism reprogramming has been identified as one of the hallmarks of malignant tumors [17], and aerobic glycolysis is the most important manifestation of energy metabolism reprogramming. Aerobic glycolysis has been demonstrated to play important roles in tumor cell proliferation, metastasis, drug resistance, and immune evasion. Previous studies reported that c-Myc was an important glycolysis-promoting transcription factor, which can upregulate target genes related to glycolysis [18–20]. To verify whether UBTD1 affects CRC metabolism, we performed UPLC-MS/MS-based metabonomics in UBTD1-overexpressing and control HCT116 stable cells. Principal component analysis (PCA) showed there was a significant difference in principal component 1 between the UBTD1 overexpression group and the control group (Fig. 4A). By analyzing the relative abundance of various metabolites in two groups of cells, it was found that the proportion of organic acids in UBTD1 overexpression cells was significantly increased (Supplementary Fig. 4A). Volcano plot and Z score plot based on metabonomics showed 54 metabolites were significantly increased and 38 metabolites were significantly decreased in UBTD1 overexpression cells (*P* < 0.05) (Supplementary Fig. 4B, C). The combined analysis of proteomics and metabonomics revealed that central carbon metabolism in cancer was the pathway with the most common participation of differential proteins and metabolites (Supplementary Fig. 4D). Specifically, compared to control cells, UBTD1 overexpression cells displayed enhanced glycolysis, as intermediate products and end-product of glycolysis were significantly increased in UBTD1 overexpression cells (Fig. 4B). To further study the function of UBTD1 in regulating aerobic glycolysis of CRC cells, we examined whether UBTD1 alteration affected glucose uptake and lactate production. We observed that overexpression of UBTD1 increased glucose uptake and lactate production, whereas UBTD1 knockdown had the opposite effect (Fig. 4C). We determined whether UBTD1 overexpression or knockdown affected the expression of various genes involved in glycolysis. We found that UBTD1 overexpression upregulated HK2 protein, which is the first rate-limiting enzyme in glycolysis, while UBTD1 knockdown downregulated HK2 (Fig. 4D). Meanwhile, UBTD1 overexpression also upregulated HK2 mRNA, whereas UBTD1 knockdown had the opposite effect (Supplementary Fig. 5). In order to verify whether UBTD1 promotes glycolysis by regulating HK2 expression, we constructed HK2 interfering plasmid, and knocked down HK2 expression in UBTD1 overexpressing HCT116 cells (Fig. 4E). HK2 knockdown could reverse the increased lactate production and glucose uptake after UBTD1 overexpression (Fig. 4F). WB and qPCR analysis revealed that c-Myc alteration affected HK2 expression in HCT116 and P53R CRC cells (Fig. 4G). In addition, the decreased expression of HK2 by UBTD1 knockdown could be rescued by c-Myc overexpression (Fig. 4H). These data suggest that UBTD1 regulates HK2 expression via c-Myc, thus promoting aerobic glycolysis in CRC.

### UBTD1 promotes CRC cell proliferation and migration via regulating glycolysis

**Fig. 5:**
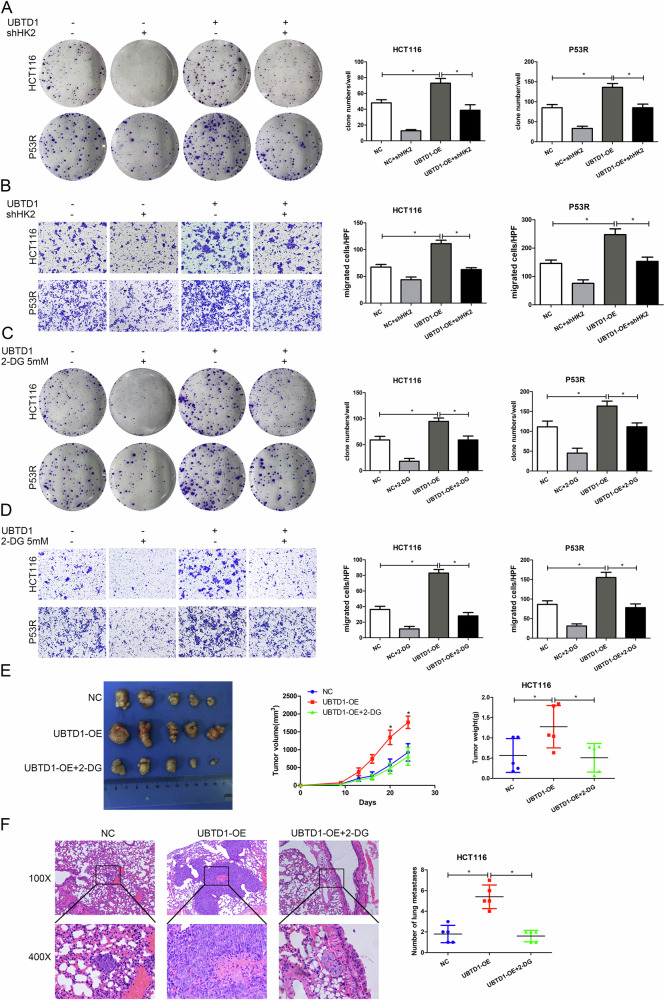
UBTD1 promotes colorectal cancer cell proliferation and migration via regulating glycolysis in vitro and in vivo. **A**, **B** Increased proliferation and migration ability in UBTD1 overexpression CRC cells can be reversed by HK2 knockdown in HCT116 and P53R cells via colony formation assay and transwell chamber assay. **C**, **D** Increased proliferation and migration ability in UBTD1 overexpression CRC cells can be reversed by treatment of 2-DG, a specific glycolysis inhibitor, in HCT116 and P53R cells via colony formation assay and transwell chamber assay. **E** Promotion of xenograft tumor growth caused by UBTD1 overexpression can be reversed by 2-DG treatment in nude mice. **F** Promotion of lung metastasis caused by UBTD1 overexpression can be reversed by 2-DG treatment in nude mice. *P < 0.05 indicates a significant difference between the indicated groups.

In order to verify whether UBTD1 promotes CRC proliferation and migration via regulating glycolysis, we repressed glycolysis in UBTD1 overexpressing HCT116 cells with glycolysis-specific inhibitor 2-DG and HK2 interfering plasmid. We revealed that the augmented proliferation and migration in UBTD1 overexpressing CRC cells were diminished after being treated with 2-DG or HK2 interfering plasmid respectively (Fig. 5A–D). In vivo, it was further demonstrated that UBTD1 overexpression CRC cells could form bigger tumors and had more metastatic lesions compared with the control cells. After being treated with 2-DG, the enhanced capacity of pro-growth and pro-metastasis in UBTD1 overexpressing CRC cells were also diminished significantly in the xenograft mouse model (Fig. 5E, F).

### UBTD1 stabilizes c-Myc protein by binding to β-TrCP and modulating c-Myc degradation

**Fig. 6:**
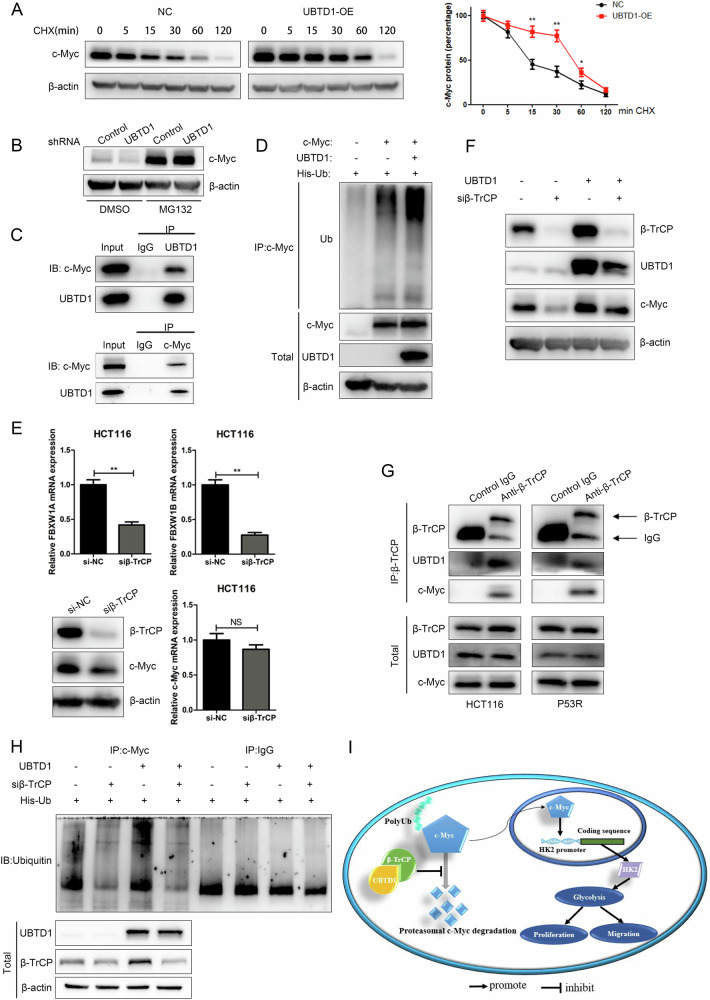
UBTD1 stabilizes c-Myc protein by interacting with β-TrCP to modulate c-Myc degradation. **A** The half-life of c-Myc protein was significantly prolonged after UBTD1 overexpression in HCT116 cells. **B** The effect of UBTD1 knockdown on c-Myc protein level was abolished after inhibition of proteasomal degradation by MG132. **C** Co-immunoprecipitation assay verified the interaction between UBTD1 and c-Myc protein. **D** In vivo ubiquitination assay revealed that UBTD1 overexpression markedly increased c-Myc ubiquitination. **E** Construction and validation of plasmids to knockdown β-TrCP, and the knockdown of β-TrCP downregulated c-Myc protein in HCT116 cells while having minimal and non-significant effect on c-Myc mRNA level. **F** Upregulation of c-Myc protein caused by UBTD1 overexpression can be attenuated by β-TrCP knockdown in HCT116 cells. **G** Co-immunoprecipitation assay revealed direct interaction among β-TrCP, UBTD1, and c-Myc in HCT116 and P53R cells. **H** β-TrCP knockdown could reverse enhanced c-Myc ubiquitination after UBTD1 overexpression. **I** The proposed working model is how UBTD1 is involved in CRC proliferation and migration. *P < 0.05 or **P < 0.01 indicates a significant difference between the indicated groups.

Ubiquitin-proteasome system (UPS) mediated targeted degradation is the major way that controls c-Myc protein level in various types of cells [21]. Since previous studies demonstrated that UBTD1 is involved in UPS-mediated protein degradation, and UBTD1 was found to regulate c-Myc protein level but not mRNA level in our study, we speculate that UBTD1 may regulate c-Myc protein by affecting the UPS-mediated c-Myc degradation in CRC. We firstly detected the effect of UBTD1 on the half-life of c-Myc protein, and found that the half-life of c-Myc protein was significantly prolonged after UBTD1 overexpression (Fig. 6A). Meantime, the effect of UBTD1 on c-Myc protein disappeared after cells were treated with proteasome inhibitor MG132 (Fig. 6B), which indicated that UBTD1 regulated c-Myc protein through ubiquitin proteasome pathway. Furtherly, co-immunoprecipitation verified the interaction between UBTD1 and c-Myc (Fig. 6C), and in vivo ubiquitination assay revealed that UBTD1 overexpression markedly increased c-Myc ubiquitination (Fig. 6D). Generally, ubiquitination leads to protein degradation, but sometimes there are exceptions. Several E3 ligases mediated c-Myc ubiquitination have been demonstrated to decrease c-Myc stability [22–25], however, the effect of β-TrCP-mediated c-Myc ubiquitination on c-Myc degradation is controversial. Popov reported that β-TrCP enhanced ubiquitination of c-Myc, but attenuated c-Myc degradation. Mechanically, β-TrCP ubiquitinates the amino terminus of c-Myc and antagonizes Fbw7-mediated c-Myc degradation [26]. However, another study demonstrated that β-TrCP promoted c-Myc ubiquitination and reduced c-Myc protein levels in nasopharyngeal carcinoma [27]. Therefore, we speculate that the effect of β-TrCP on c-Myc is complicated. Interestingly, previous studies have discovered that UBTD1 regulates the ubiquitination of substrate proteins by promoting the association of substrate proteins and β-TrCP [7, 8]. In our study, we found that knockdown of β-TrCP in CRC cells downregulated c-Myc protein and overexpression of β-TrCP had the opposite effect, but β-TrCP had no effect on c-Myc mRNA (Fig. 6E and Supplementary Fig. 6A), which is consistent with Popov’s study [26]. To explore whether UBTD1 regulates c-Myc protein level via β-TrCP, we interfered with β-TrCP expression in UBTD1 overexpressing CRC cells, and found that β-TrCP interfering could reverse the increased level of c-Myc protein caused by UBTD1 overexpression (Fig. 6F). Meanwhile, UBTD1 overexpression was able to restore c-Myc downregulation caused by Fbw7 overexpression, while the mutated-UBTD1 couldn’t (Supplementary Fig. 6C), indicating that intact ubiquitin domain was critical for UBTD1 in helping β-TrCP to ubiquitinate and stabilize c-Myc, competitively against the ubiquitination of Fbw7 on c-Myc protein. Interestingly, we found that overexpression of UBTD1 upregulated β-TrCP, and knocking down UBTD1 had the opposite effect (Fig. 6F lane 1 and lane 3, and Supplementary Fig. 6B). The underlying mechanism remains to be elucidated. Further, we used Co-IP assay and confirmed the interaction between β-TrCP, UBTD1, and c-Myc (Fig. 6G). Additionally, in vivo ubiquitination assay showed that β-TrCP knockdown could reverse enhanced c-Myc ubiquitination after UBTD1 overexpression (Fig. 6H), which indicated β-TrCP is indispensable for UBTD1-mediated ubiquitination of c-Myc. In conclusion, our results revealed that UBTD1 enhanced glycolysis to promote CRC progression through stabilizing c-Myc protein by interacting with E3 ligase β-TrCP (Fig. 6I).

## Discussion

The ubiquitin-like protein UBTD1 plays a pivotal role in the degradation of key proteins in various cancer cells by interacting with E3 ligases, significantly influencing cancer progression and invasion [7–10]. In our study, we discovered that UBTD1 interacts with the E3 ligase β-TrCP, stabilizing the c-Myc oncoprotein. This interaction enhances glycolysis in colorectal cancer (CRC) cells through the c-Myc/HK2 signaling pathway, ultimately amplifying the malignant behavior of these cells (Fig. 6I, Schematic model).

Proteins are the main executors of biological processes intracellularly. As one of the main approaches to degrade intracellular protein in humans, ubiquitination is an essential posttranslational modification which can degrade, stable, or relocate the target proteins by covalently attaching with ubiquitin. Three classes of enzymes (E1, ubiquitin-activating enzymes, E2, ubiquitin-conjugating enzymes, E3, ubiquitin ligases) are involved in the ubiquitination process. E1 enzymes activate ubiquitin with the aid of adenosine triphosphate (ATP) and transfer activated ubiquitin to E2, then E3 attaches the activated ubiquitin-E2 complex to the substrate proteins [28]. The UPS is well recognized for its pivotal role in regulating the degradation of oncoproteins, thereby maintaining cellular homeostasis [5, 29].

*UBTD1* gene, located in chromosome 10, is highly evolutionarily conserved and encodes a ubiquitin-like protein featuring a ubiquitin domain in its C terminal [6]. While some ubiquitin-like proteins lack the ability to directly modify target proteins, they are often pivotal in regulating the modification that impacts the stability and activity of these proteins [30, 31]. Previous studies found that UBTD1 can interact with E3 ligase β-TrCP to regulate the ubiquitination of oncoprotein YAP, leading to the degradation of YAP and suppression of cancer cells’ malignant phenotype [7, 8]. An additional study reported that UBTD1 can interact with another E3 ligase, RNF26, to upregulate the ubiquitination of autophagy-related protein p62 and enhance the lysosomal degradation of EGFR in prostate cancer cells [9]. In our current study, it was discovered that the UBTD1/β-TrCP complex could stabilize the c-Myc protein, contrasting with its previously reported effects on YAP. The impact of β-TrCP on c-Myc stability remains a subject of debate [26, 27], with the different effects potentially attributed to distinct proteins or RNAs interacting with β-TrCP. Further studies are warranted to elucidate the mechanisms behind these varying effects of β-TrCP on c-Myc stability. Besides, several studies have indicated that β-TrCP can function as either a tumor suppressor or oncoprotein, depending on the ubiquitination of different target proteins across different tumor types [32–35]. In CRC, β-TrCP has been identified as an oncoprotein and a biomarker for tumor recurrence and poor prognosis [36, 37].

In our current study, we discovered that UBTD1 binds with β-TrCP through Co-IP assay. Proteomics analysis revealed that c-Myc was the most significant target protein of UBTD1, standing out among over 6000 detectable proteins. Meanwhile, we observed that the MYC pathway was significantly enriched in UBTD1 overexpressed CRC cells through GSEA, but the c-Myc mRNA level was not significantly impacted in both RNA-seq and qRT-PCR analysis. Additionally, we verified that β-TrCP knockdown could downregulate c-Myc at the protein level, and overexpression of UBTD1 cannot upregulate c-Myc at the protein level in β-TrCP depleted CRC cells, indicating that β-TrCP was essential for UBTD1 to upregulate c-Myc protein. We further demonstrated that UBTD1 could enhance the ubiquitination of c-Myc protein in CRC cells. Previous studies have reported that β-TrCP could stabilize c-Myc protein by antagonizing Fbw7-mediated turnover of c-Myc via ubiquitinating its C terminal to form atypical chains of ubiquitin [26]. Collectively, our findings demonstrated that UBTD1 could upregulate the c-Myc protein by interacting with β-TrCP.

The degradation of c-Myc protein was intricately regulated by a sophisticated network, involving dozens of E3 ligases and de-ubiquitin enzymes [21]. Within the network, the β-TrCP and FBW7 proteins play essential but opposing functions in the regulation of c-Myc stability. FBW7 is known to ubiquitinate c-Myc c-terminal to form K48-linked ubiquitin chains and lead to c-Myc degradation. In our study, we found that β-TrCP was the major player for c-Myc ubiquitination, promoting the ubiquitination of c-Myc, and thereby enhancing its stability. We additionally found that UBTD1 could facilitate this process by interacting with β-TrCP. This interaction may provide insight into the dual role of UBTD1, which has been characterized as a tumor suppressor in some studies but behaves as an oncoprotein in CRC cells.

Besides, our research discovered that UBTD1 mainly upregulates the glucose metabolism pathway in CRC cells, as revealed by KEGG analysis of the proteomics data. Subsequent to this discovery, we verified that UBTD1 could upregulate glycolysis in CRC cells via metabonomic analysis and in vitro experiments. Abnormal glucose metabolism is a hallmark of tumor cells. The intricate interplay between metabolism and cancer development has emerged as a pivotal area of research in oncology. Cancer cells often exhibit a reprogrammed metabolism, characterized by increased glucose uptake and fermentation of pyruvate to lactate, even in the presence of oxygen, a phenomenon known as the Warburg effect [38]. The metabolic reprogramming supports the elevated biosynthetic demands of rapidly proliferating cancer cells, providing them with the necessary building blocks for cell growth and division. Moreover, metabolic alterations can influence the tumor microenvironment, potentially promoting cancer progression and metastasis. Understanding these metabolic pathways offers promising avenues for the development of novel therapeutic strategies targeting cancer metabolism. Importantly, glycolysis has been demonstrated to play significant roles in tumor growth, metastasis, drug resistance, immune escape, et al [39–42]. In our study, increased proliferation and metastasis of CRC cells after UBTD1 overexpression could be reversed by a specific glycolysis inhibitor 2-DG in vitro and in vivo, which indicated that UBTD1 regulated CRC cell's malignant phenotype via promoting glycolysis.

Recent studies have revealed that the tumor reprogramming of metabolism is associated with the persistent high expression of glycolysis-related genes, with HIF-1α and c-Myc are considered as the most critical glycolysis-promoting transcription factors [43]. In our study, we screened many glycolysis-related genes (HK1, HK2, LDHA, LDHB, ALDOA, and GLUT3), and identifed HK2 as a downstream effector gene for the enhanced glycolysis, which was upregulated by UBTD1 overexpression in CRC cells. Overexpression of c-Myc could rescue HK2 expression in UBTD1 knockdown CRC cell lines, indicating UBTD1 could promote HK2 expression via upregulating c-Myc. Generally, these findings profoundly demonstrate that the UBTD1/β-TrCP complex ubiquitinates c-Myc protein, then stabilize it and lead to the upregulation of HK2, finally promote the glycolysis in CRC cells and lead to growth and migration.

Bioinformatic analysis showed that UBTD1 expression was significantly higher in CRC tissues than in adjacent normal tissues, and higher UBTD1 was associated with poorer prognosis using multivariate Cox regression analysis. These findings indicated that UBTD1 may serve as a potentially important and independent prognosis biomarker in CRC. However, the clinical data which were leveraged in this research is retrospective and the analysis was conducted in limited sample size, statistical conclusion should be interpreted with caution, and confirmation through prospective large cohort studies are warranted to validate these preliminary insights.

Ubiquitin-like proteins, as important component of UPS degradation pathway, have been widely investigated in the pharmaceutical area. The β-TrCP and VHL protein, as important E3 enzymes, have been used as important degraders in PROTAC technique to target some proteins that are undruggable by traditional methods [44, 45]. Further studies regarding of UBTD1/β-TrCP complex may provide novel target for CRC treatments.

### Availability of data and materials

The FUSCC datasets used and/or analyzed, or R language code during the current study are available from the corresponding author upon reasonable request.

### Supplementary information

Supplementary table 1

Supplementary table 2

Supplementary table 3

Supplementary table 4

Supplementary figure legend

supplementary figure 1

supplementary figure 2

supplementary figure 3

supplementary figure 4

supplementary figure 5

supplementary figure 6

uncropped original western blots
