# Supplementary material for: The ubiquitin-like protein UBTD1 promotes colorectal cancer progression by stabilizing c-Myc to upregulate glycolysis
Source: Cell Death Dis. 2024 Jul 13;15(7):502. doi: 10.1038/s41419-024-06890-5 (PMC11246417; doi:10.1038/s41419-024-06890-5)
Supplement: Supplementary file 1 — Supplementary table 1 [file 41419_2024_6890_MOESM1_ESM.docx]

| **Table S1.** Sequence of primers that are used in our study. | |
| --- | --- |
| Primer name | Sequence（5’-3’） |
| GAPDH-Forward | ACCCAGAAGACTGTGGATGG |
| GAPDH-Reverse | TTCTAGACGGCAGGTCAGGT |
| UBTD1-Forward | ACAGTGGGGCAGCTCAAGAGG |
| UBTD1-Reverse | TTGGTCTCCTGGAGCCGTGTG |
| c-Myc-Forward | GGCTCCTGGCAAAAGGTCA |
| c-Myc-Reverse | CTGCGTAGTTGTGCTGATGT |
| HK2-Forward | CGACAGCATCATTGTTAAGGAG |
| HK2-Reverse | GCAGGAAAGACACATCACATTT |
| FBXW1A-Forward | TGCTCTATGCCCAGGTCTCT |
| FBXW1A-Reverse | AGGGGGTTCGCCATTATTAC |
| FBXW1B-Forward | AAACCAGCCTGGAATGTTTG |
| FBXW1B-Reverse | CAGTCCATTGCTGAAGCGTA |
