# Supplementary material for: The ubiquitin-like protein UBTD1 promotes colorectal cancer progression by stabilizing c-Myc to upregulate glycolysis
Source: Cell Death Dis. 2024 Jul 13;15(7):502. doi: 10.1038/s41419-024-06890-5 (PMC11246417; doi:10.1038/s41419-024-06890-5)
Supplement: Supplementary file 2 — Supplementary table 2 [file 41419_2024_6890_MOESM2_ESM.docx]

| **Table S2.** Clinicopathologic characteristics of TGCA COAD and READ Patients With high UBTD1 expression vs low UBTD1 expression. | | | |
| --- | --- | --- | --- |
| Characteristics | No. of Cases (%) | | |
|  | High | Low | *P* Value |
| All Subjects | 295 (100) | 294 (100) |  |
| Age (mean (SD)) | 66.32 (13.06) | 65.98 (12.68) | 0.747 |
| Gender |  |  |  |
| Female | 134 (45.4) | 145 (49.3) | 0.387 |
| Male | 161 (54.6) | 149 (50.7) |  |
| Cancer types |  |  | 0.87 |
| COAD | 217 (73.6) | 219 (74.5) |  |
| READ | 78 (26.4) | 75 (25.5) |  |
| T stage |  |  |  |
| T1-T2 | 49 (16.6) | 74 (25.2) | **0.014** |
| T3-T4 | 246 (83.4) | 220 (74.8) |  |
| N stage |  |  |  |
| N negative | 151 (51.2) | 188 (63.9) | **0.003^a)^** |
| N positive | 143 (48.5) | 106 (36.1) |  |
| Missing | 1 (0.3) | 0 (0) |  |
| M stage |  |  |  |
| M0 | 212 (71.9) | 226 (76.9) | **0.03^a)^** |
| M1 | 51 (17.3) | 31 (10.5) |  |
| Missing | 32 (10.8) | 37 (12.6) |  |
| TNM stage |  |  |  |
| Stage I-II | 144 (48.8) | 178 (60.5) | **0.003^a)^** |
| Stage III-IV | 147 (49.9) | 108 (36.8) |  |
| Missing | 4 (1.4) | 8 (2.7) |  |
| Radiation therapy |  |  | 0.603 |
| Yes | 14 (4.7) | 13 (4.4) |  |
| No | 235 (79.7) | 226 (76.9) |  |
| NA | 46 (15.6) | 55 (18.7) |  |
| **Abbreviations:** TCGA, The Cancer Genome Atlas; COAD, colon adenocarcinoma; READ, rectal adenocarcinoma; UBTD1: ubiquitin domain containing 1; and SD: standard deviation.  The results were in **bold**, if the *P* value was less than 0.05.  a) Excluding those with missing data. | | | |
