# Supplementary material for: The ubiquitin-like protein UBTD1 promotes colorectal cancer progression by stabilizing c-Myc to upregulate glycolysis
Source: Cell Death Dis. 2024 Jul 13;15(7):502. doi: 10.1038/s41419-024-06890-5 (PMC11246417; doi:10.1038/s41419-024-06890-5)
Supplement: Supplementary file 3 — Supplementary table 3 [file 41419_2024_6890_MOESM3_ESM.docx]

| **Table S3.** Clinicopathologic characteristics of FUSCC CRC patients with high UBTD1 expression and low UBTD1 expression. | | | |
| --- | --- | --- | --- |
|  | UBTD1 expression level | |  |
| Features | Low, 44(100%) | High, 75 (100%) | *P* Value |
| Gender |  |  | 0.358 |
| Female | 21 (47.7) | 28 (37.3) |  |
| Male | 23 (52.3) | 47 (62.7) |  |
| Age |  |  | 0.509 |
| >65 | 27 (61.4) | 40 (53.3) |  |
| ≤65 | 17 (38.6) | 35 (46.7) |  |
| TNM Stage |  |  | 0.177 |
| Stage Ⅰ | 10 (22.7) | 10 (13.3) |  |
| Stage Ⅱ | 13 (29.5) | 27 (36.0) |  |
| Stage Ⅲ | 12 (27.3) | 30 (40.0) |  |
| Stage Ⅳ | 9 (20.5) | 8 (10.7) |  |
| Subtype |  |  | 0.273 |
| COAD | 26 (59.1) | 51 (68.0) |  |
| READ | 11 (25.0) | 10 (13.3) |  |
| Unknown | 7 (15.9) | 14 (18.7) |  |
| Histological differentiation |  |  | 0.709 |
| Well | 5(11.4) | 5(6.7) |  |
| Moderate | 28(63.6) | 53(70.6) |  |
| Poor | 10(22.7) | 14(18.7) |  |
| Unknown | 1(2.3) | 3(4.0) |  |
| Abbreviations: FUSCC, Fudan University Shanghai Cancer Center; CRC, colorectal cancer; COAD, Colon Adenocarcinoma; READ, Rectal Adenocarcinoma. *P* < 0.05 is statistically significant | | | |
