# Supplementary material for: The ubiquitin-like protein UBTD1 promotes colorectal cancer progression by stabilizing c-Myc to upregulate glycolysis
Source: Cell Death Dis. 2024 Jul 13;15(7):502. doi: 10.1038/s41419-024-06890-5 (PMC11246417; doi:10.1038/s41419-024-06890-5)
Supplement: Supplementary file 4 — Supplementary table 4 [file 41419_2024_6890_MOESM4_ESM.docx]

| **Table S4.** Univariate and multivariate analysis of the association between UBTD1 expression level and overall survival of colorectal cancer patients | | | | | | | |
| --- | --- | --- | --- | --- | --- | --- | --- |
| Variables | all case (%) | death case (%) | | Univariate analysis | | Multivariate analysis | |
|  | 586(100) | 118(100) | HR (95% CI) | | *P* value | HR (95%CI) | *P* value |
| Age |  |  |  | |  |  |  |
| ＜68 | 292 (49.8) | 34 (28.8) | 1.00 | | reference | 1.00 | reference |
| ≥68 | 294 (50.2) | 84 (71.2) | 2.517 (1.689-3.749) | | **5.65E-06** | 2.847 (1.9024-4.260) | **3.63E-07** |
| Gender |  |  |  | |  |  |  |
| Female | 278 (47.4) | 54 (45.8) | 1.00 | | reference | 1.00 | reference |
| Male | 308 (52.6) | 64 (54.2) | 1.119 (0.7775-1.61) | | 0.546 | 1.050 (0.7297-1.512) | 0.7918 |
| TNM stage |  |  |  | |  |  |  |
| Stage Ⅰ-Ⅱ | 326 (55.6) | 41 (34.7) | 1.00 | | reference | 1.00 | reference |
| Stage Ⅲ-Ⅳ | 260 (44.4) | 77 (65.3) | 2.871 (1.961-4.202) | | **5.78E-08** | 3.014 (2.0393-4.455) | **3.11E-08** |
| UBTD1 level |  |  |  | |  |  |  |
| ＜best cutoff | 368 (62.8) | 61 (51.7) | 1.00 | | reference | 1.00 | reference |
| ≥best cutoff | 218 (37.2) | 57 (48.3) | 1.98 (1.377-2.849) | | **0.000232** | 1.594 (1.1018-2.305) | **0.0133** |
| **Abbreviations:** UBTD1: ubiquitin domain containing 1; HR: hazard ratios; and CI: confidence interval.  The results were in **bold**, if the *P* value was less than 0.05. | | | | | | | |
