# Supplementary material for: The ubiquitin-like protein UBTD1 promotes colorectal cancer progression by stabilizing c-Myc to upregulate glycolysis
Source: Cell Death Dis. 2024 Jul 13;15(7):502. doi: 10.1038/s41419-024-06890-5 (PMC11246417; doi:10.1038/s41419-024-06890-5)
Supplement: Supplementary file 5 — Supplementary figure legend [file 41419_2024_6890_MOESM5_ESM.docx]

**Supplementary figure 1.** GSEA based on TCGA CRC datasets, UBTD1 subcellular localization and pathways enriched in UBTD1 overexpression cells based on RNA-seq. A: GSEA based on TCGA CRC datasets suggested that EMT, hypoxia, and inflammatory response pathways was significantly enriched in UBTD1-high CRC cases. B: UBTD1 mRNA level is positively correlated with CDH2, vimentin, MMP9, Snail, Slug, but negatively correlated with CDH1. C: Immunofluorescence showed UBTD1 mainly located in cytoplasm rather than nucleus. D: GSEA based on RNA-seq indicated that MYC signaling was greatly enriched in UBTD1-overexpressing cells.

**Supplementary figure 2.** Ubiquitin domain is essential for biological function of UBTD1. A: Protein-protein interaction analysis based on proteomics in UBTD1 overexpression HCT116 cells comparing to the control HCT116 cells. B: Overexpression of mutated UBTD1 that removed ubiquitin domain had on effect on c-Myc protein. C-D: Overexpression of mutated UBTD1 that removed ubiquitin domain had on effect on cell proliferation and migration. ***P*＜0.01 indicates a significant difference between the indicated groups.

**Supplementary figure 3.** Gene Ontology and KEGG pathway analysis based on proteomics. A: Gene Ontology based on proteomics revealed UBTD1 participated in metabolic process and biological regulation. B: KEGG pathway analysis indicated that UBTD1 may be involved in signal pathways related to glucose metabolism.

**Supplementary figure 4.** Analysis of metabonomics and combined analysis of proteomics and metabonomics. A: The proportion of organic acids in UBTD1 overexpression cells was significantly increased. B: Volcano plot based on metabonomics. C: Z score plot based on metabonomics. D: Combined analysis of proteomics and metabonomics.

**Supplementary figure 5.** UBTD1 could regulate HK2 mRNA level. A: UBTD1 overexpression upregulated HK2 mRNA. B: UBTD1 knockdown downregulated HK2 mRNA. **P*＜0.05 or ***P*＜0.01 indicates a significant difference between the indicated groups.

**Supplementary figure 6.** UBTD1 regulates β-TrCP expression and could restore c-Myc protein after Fbw7 overexpression. A: Overexpression of β-TrCP upregulated c-Myc protein while knockdown of β-TrCP had the opposite effect. B: Overexpression of UBTD1 upregulated β-TrCP, and knockdown of UBTD1 had the opposite effect. C: UBTD1 overexpression was able to restore c-Myc down-regulation caused by Fbw7 overexpression, while the mutated-UBTD1 couldn’t.
