# Supplementary figures and images for: The ubiquitin-like protein UBTD1 promotes colorectal cancer progression by stabilizing c-Myc to upregulate glycolysis
Source: Cell Death Dis. 2024 Jul 13;15(7):502. doi: 10.1038/s41419-024-06890-5 (PMC11246417; doi:10.1038/s41419-024-06890-5)

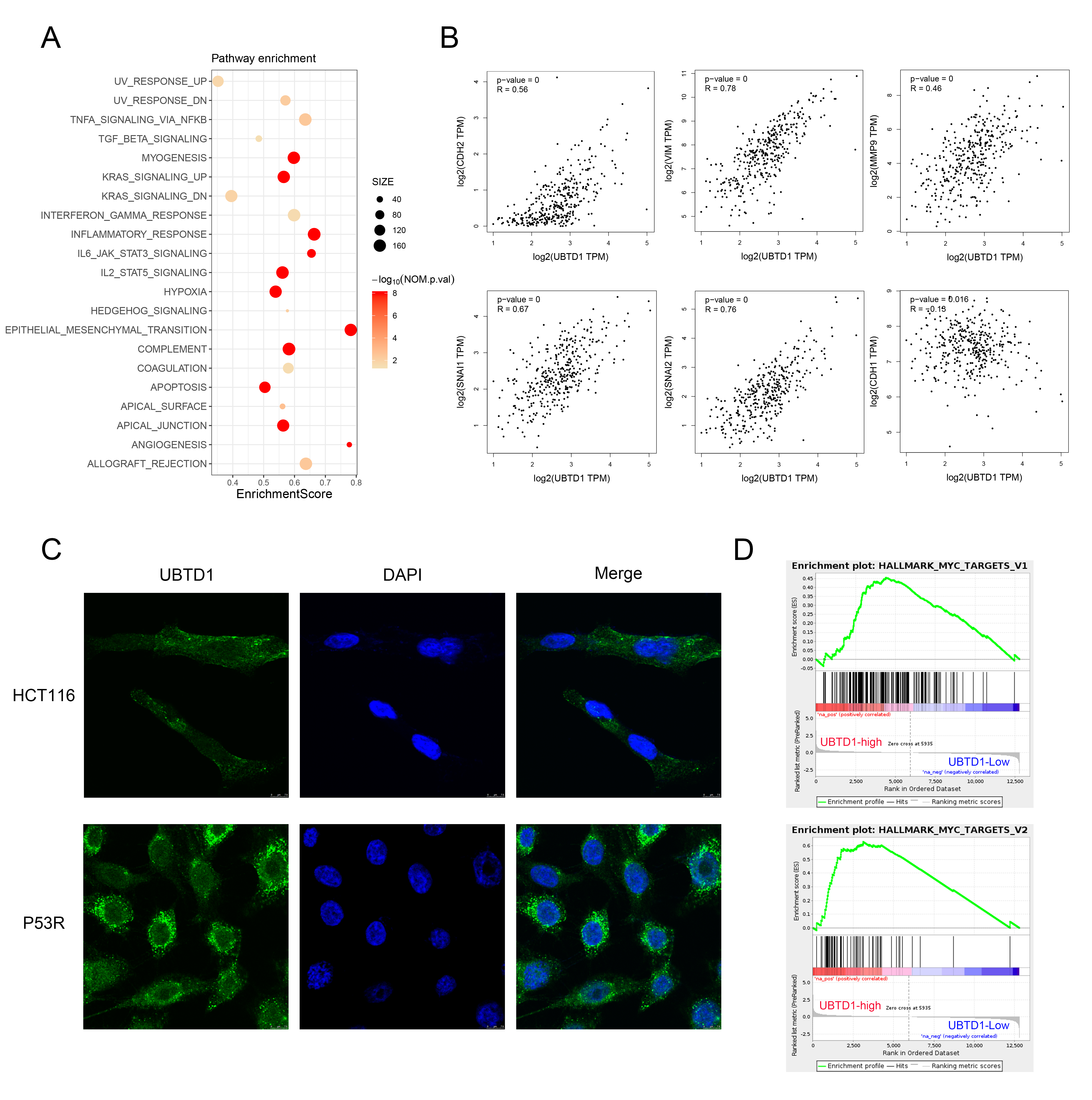

Supplement: Supplementary file 6 — supplementary figure 1 [file 41419_2024_6890_MOESM6_ESM.tif]

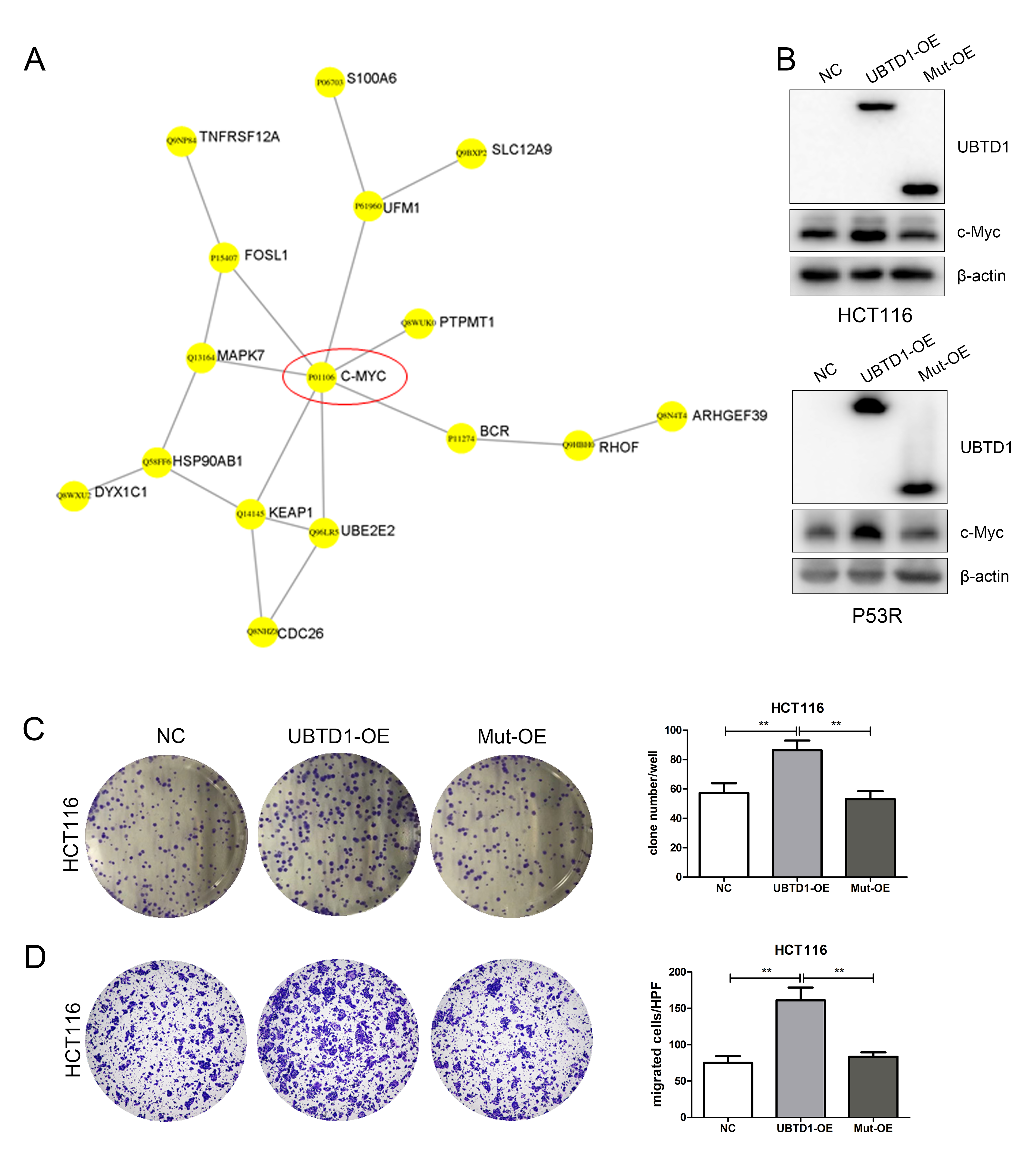

Supplement: Supplementary file 7 — supplementary figure 2 [file 41419_2024_6890_MOESM7_ESM.tif]

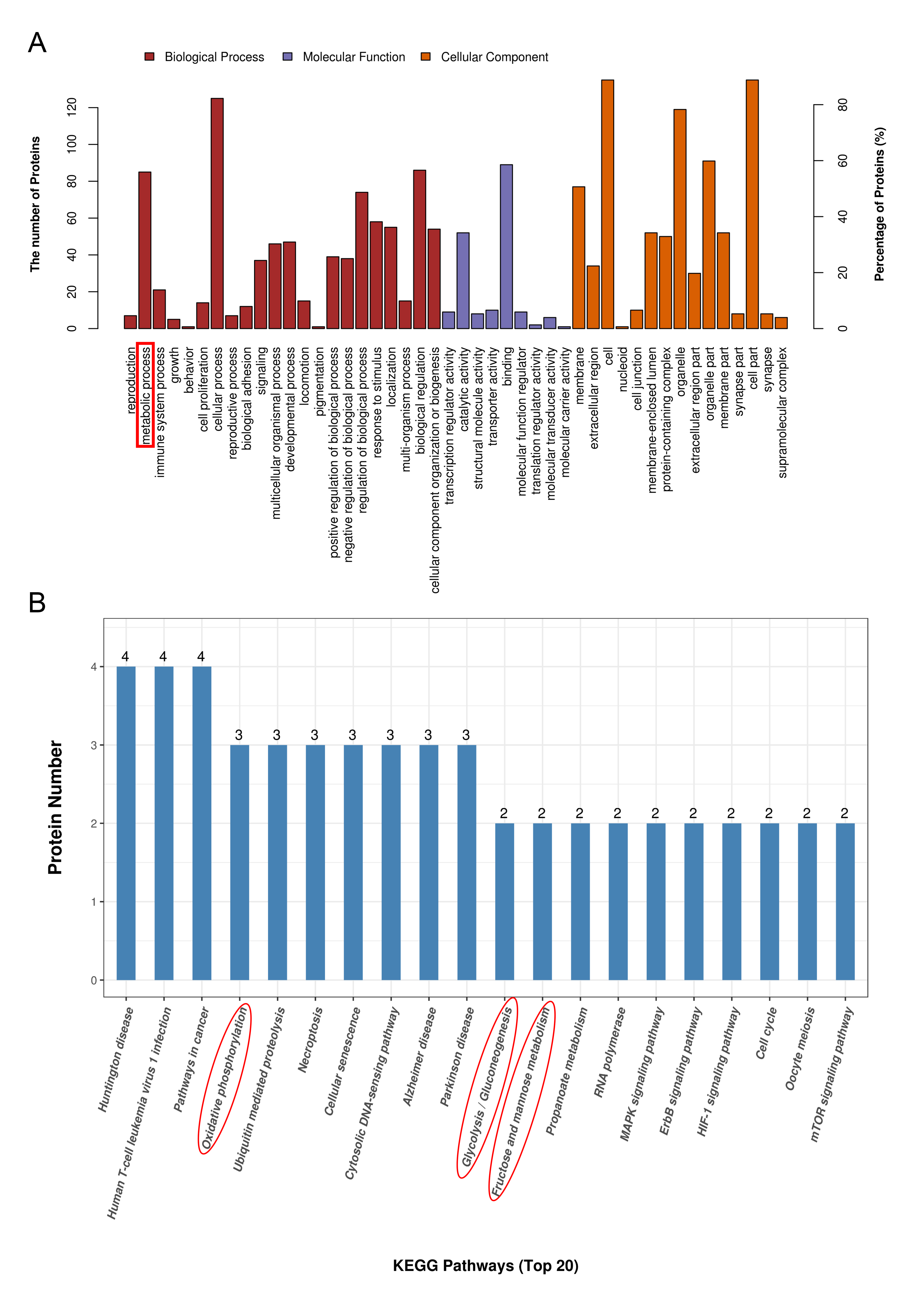

Supplement: Supplementary file 8 — supplementary figure 3 [file 41419_2024_6890_MOESM8_ESM.tif]

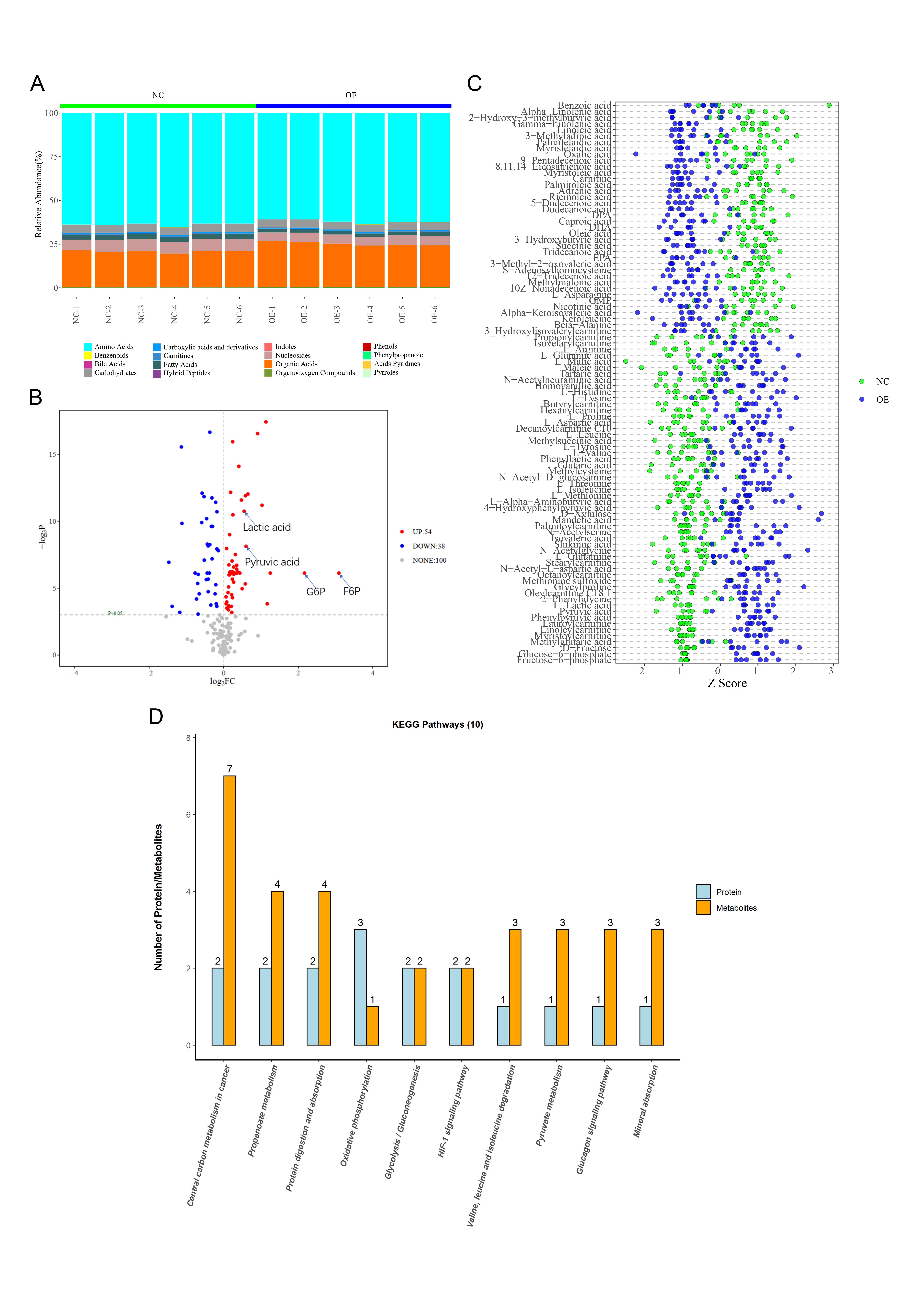

Supplement: Supplementary file 9 — supplementary figure 4 [file 41419_2024_6890_MOESM9_ESM.tif]

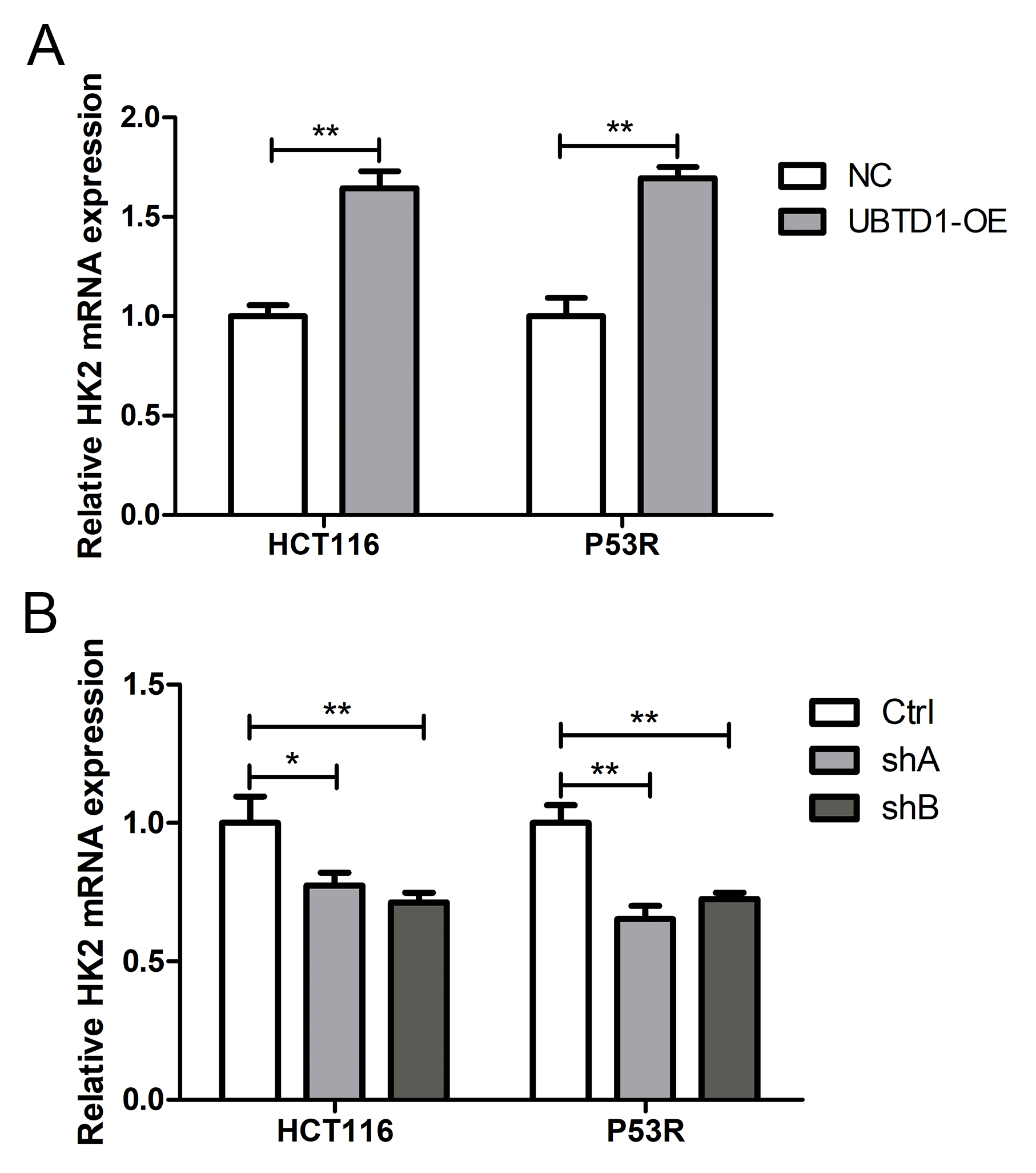

Supplement: Supplementary file 10 — supplementary figure 5 [file 41419_2024_6890_MOESM10_ESM.tif]

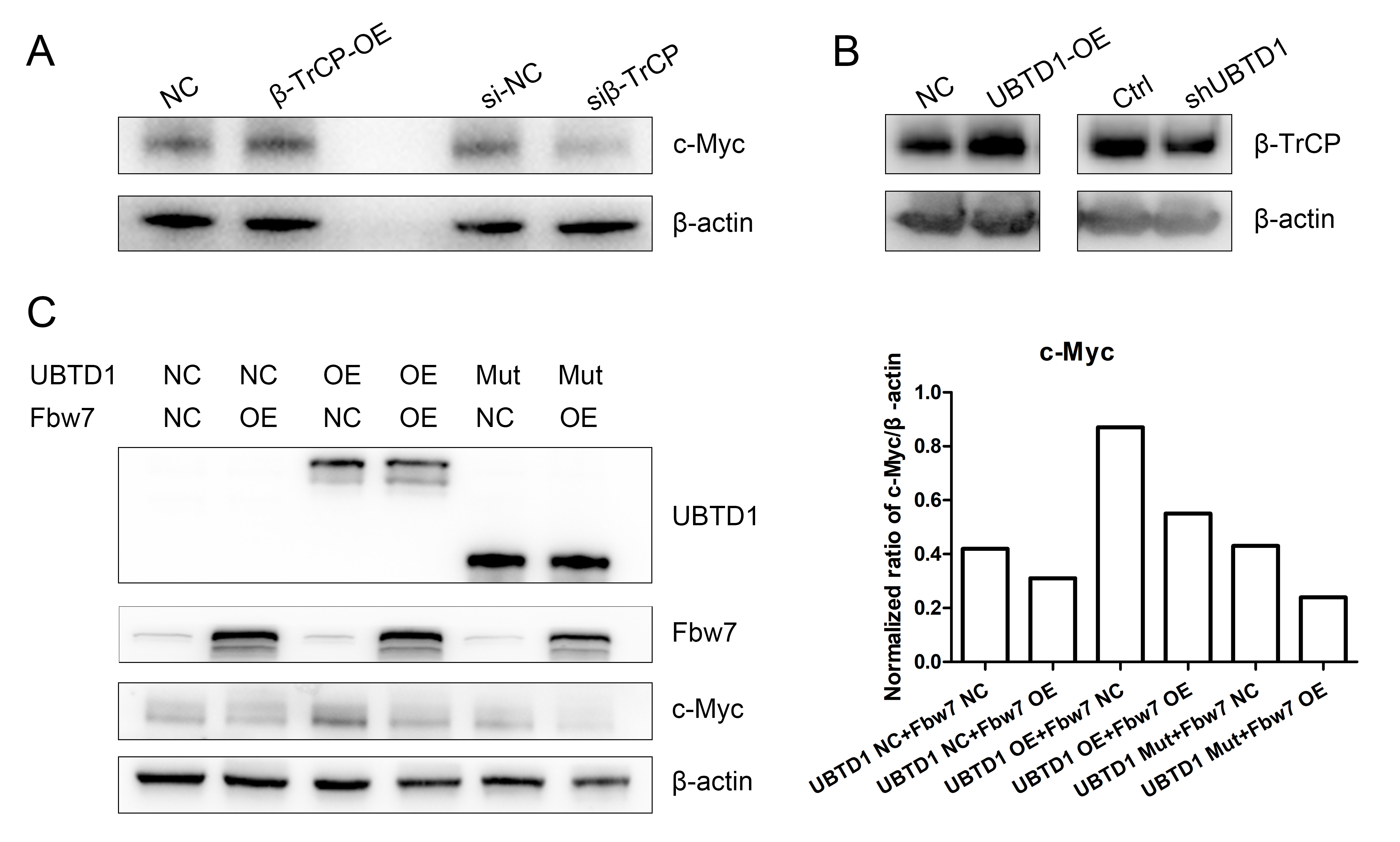

Supplement: Supplementary file 11 — supplementary figure 6 [file 41419_2024_6890_MOESM11_ESM.tif]
