# Supplementary material for: The ubiquitin-like protein UBTD1 promotes colorectal cancer progression by stabilizing c-Myc to upregulate glycolysis
Source: Cell Death Dis. 2024 Jul 13;15(7):502. doi: 10.1038/s41419-024-06890-5 (PMC11246417; doi:10.1038/s41419-024-06890-5)

Figure 1B

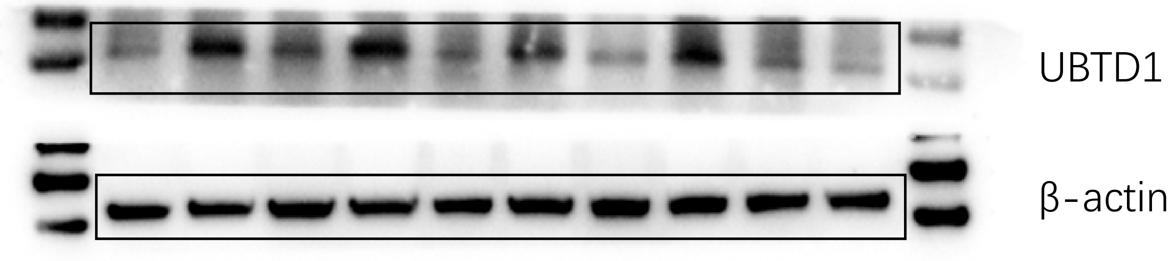

Figure 1C

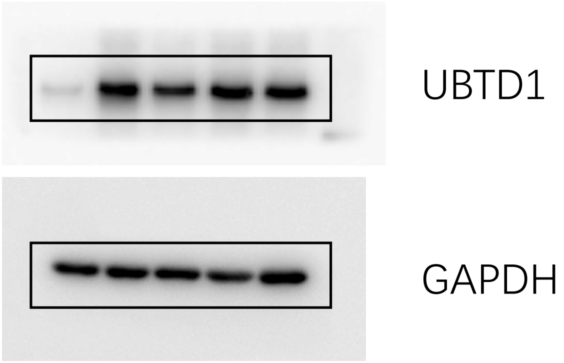

Figure 2A

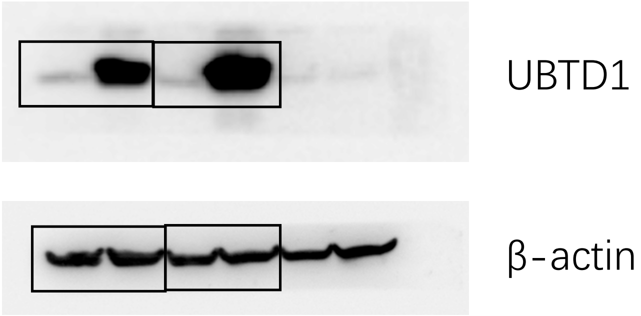

Figure 2B

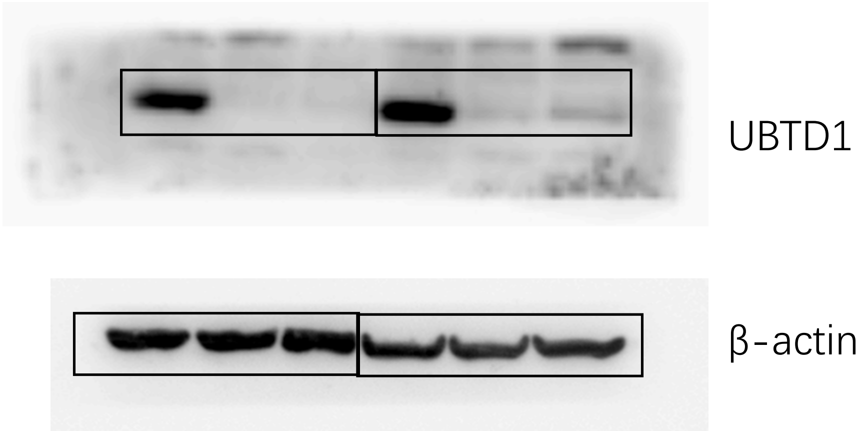

Figure 3E

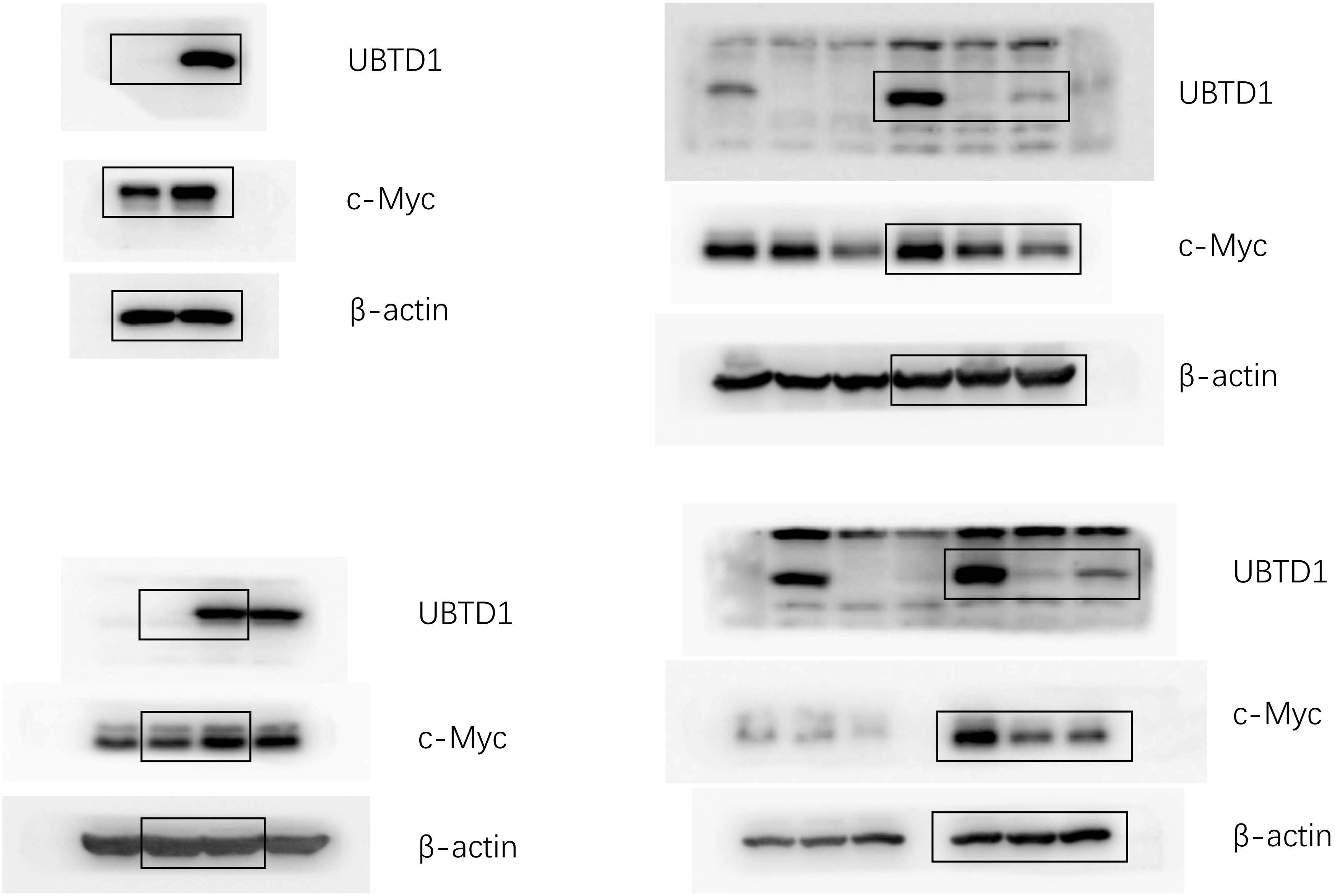
UBTD1
c-Myc
β-actin
UBTD1
c-Myc
β-actin
UBTD1
c-Myc
β-actin
UBTD1
c-Myc
β-actin

Figure 4D

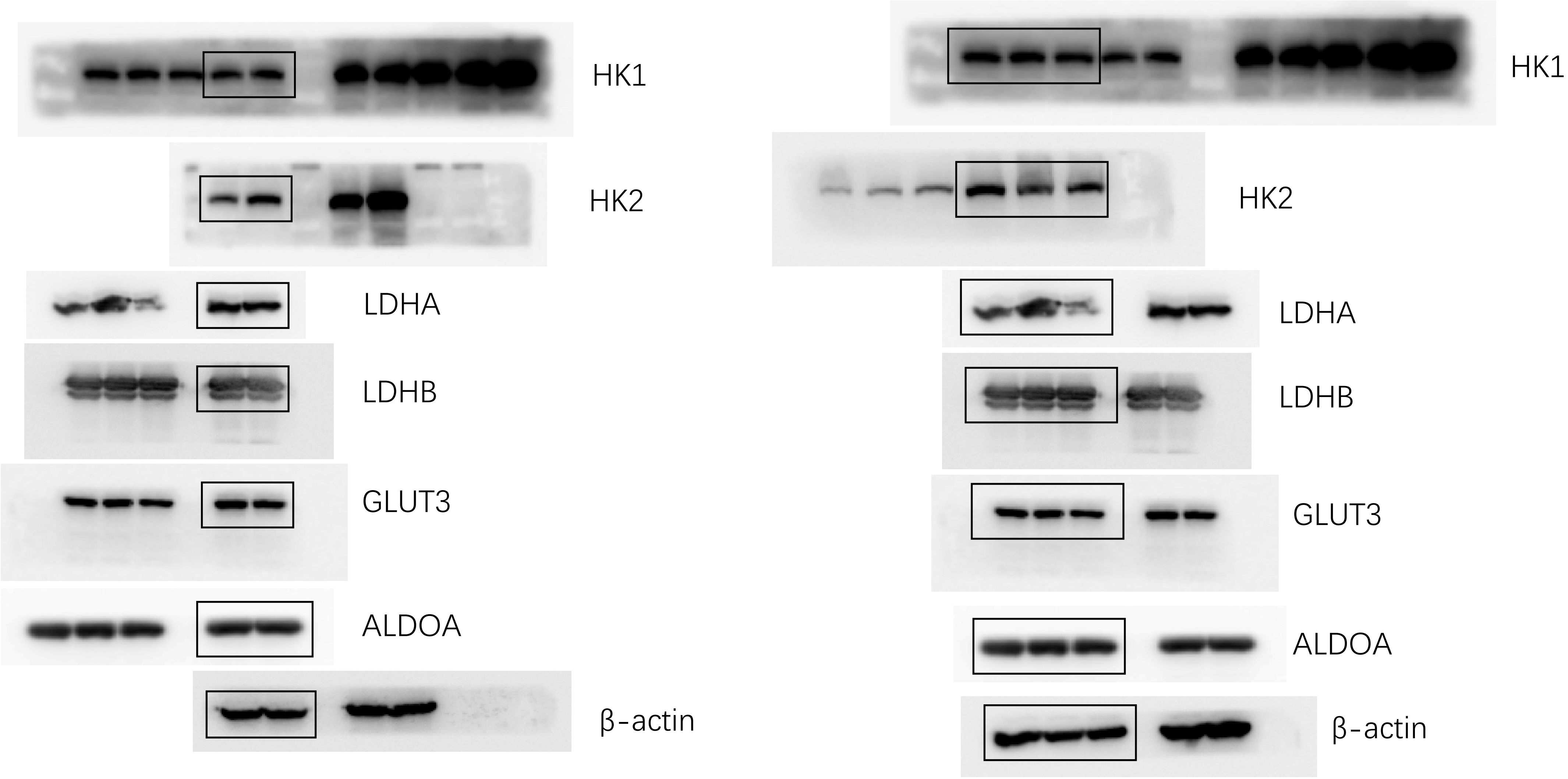
HK1
HK2
LDHA
LDHB
GLUT3
ALDOA
β-actin
HK1
HK2
LDHA
LDHB
GLUT3
ALDOA
β-actin

Figure 4D

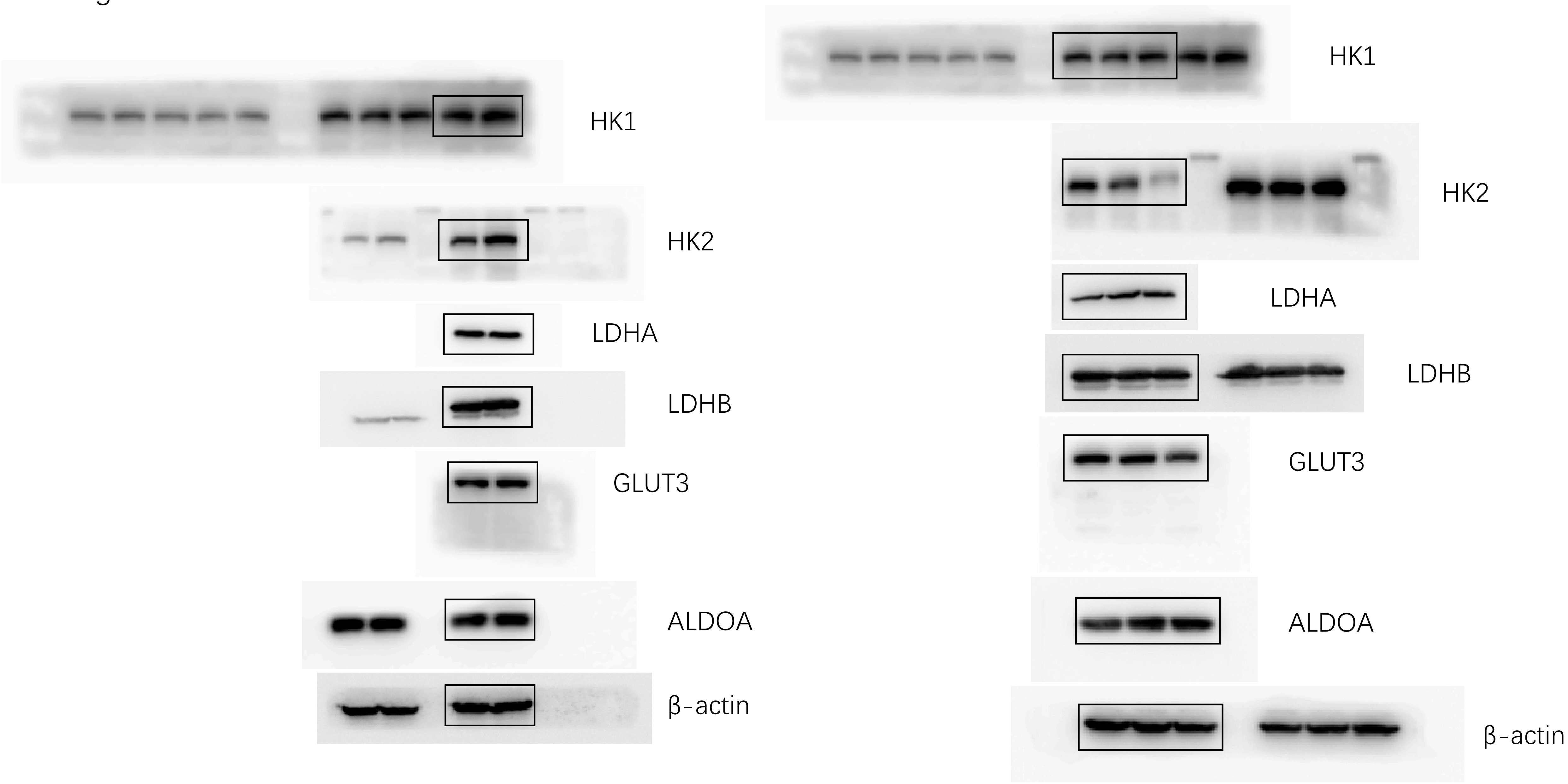
HK1
HK2
LDHA
LDHB
GLUT3
ALDOA
β-actin
HK1
HK2
LDHA
LDHB
GLUT3
ALDOA
β-actin

Figure 4E

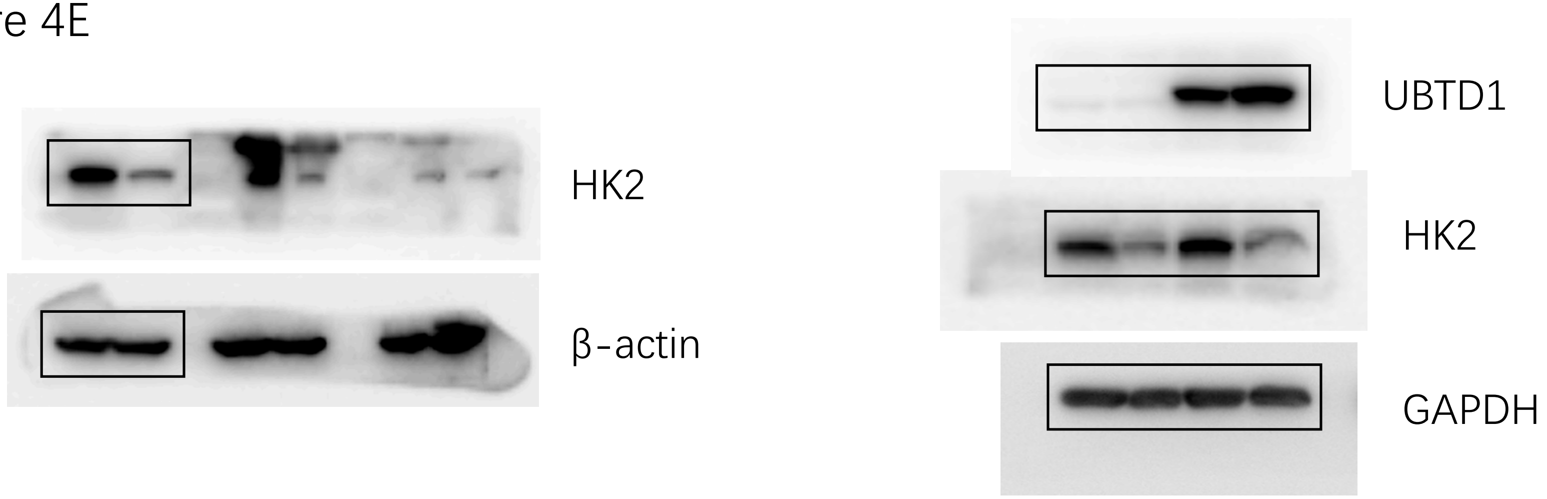

Figure 4G

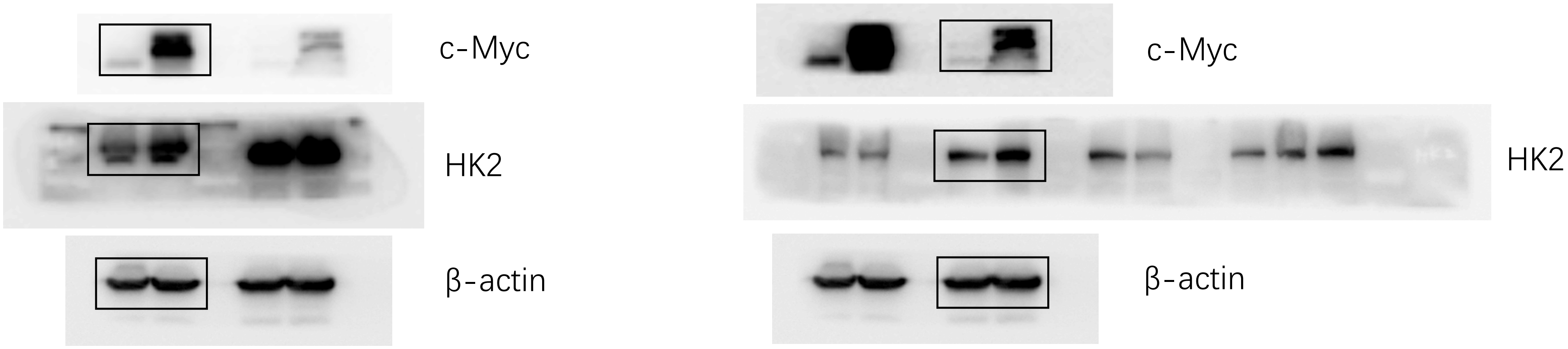

Figure 4H

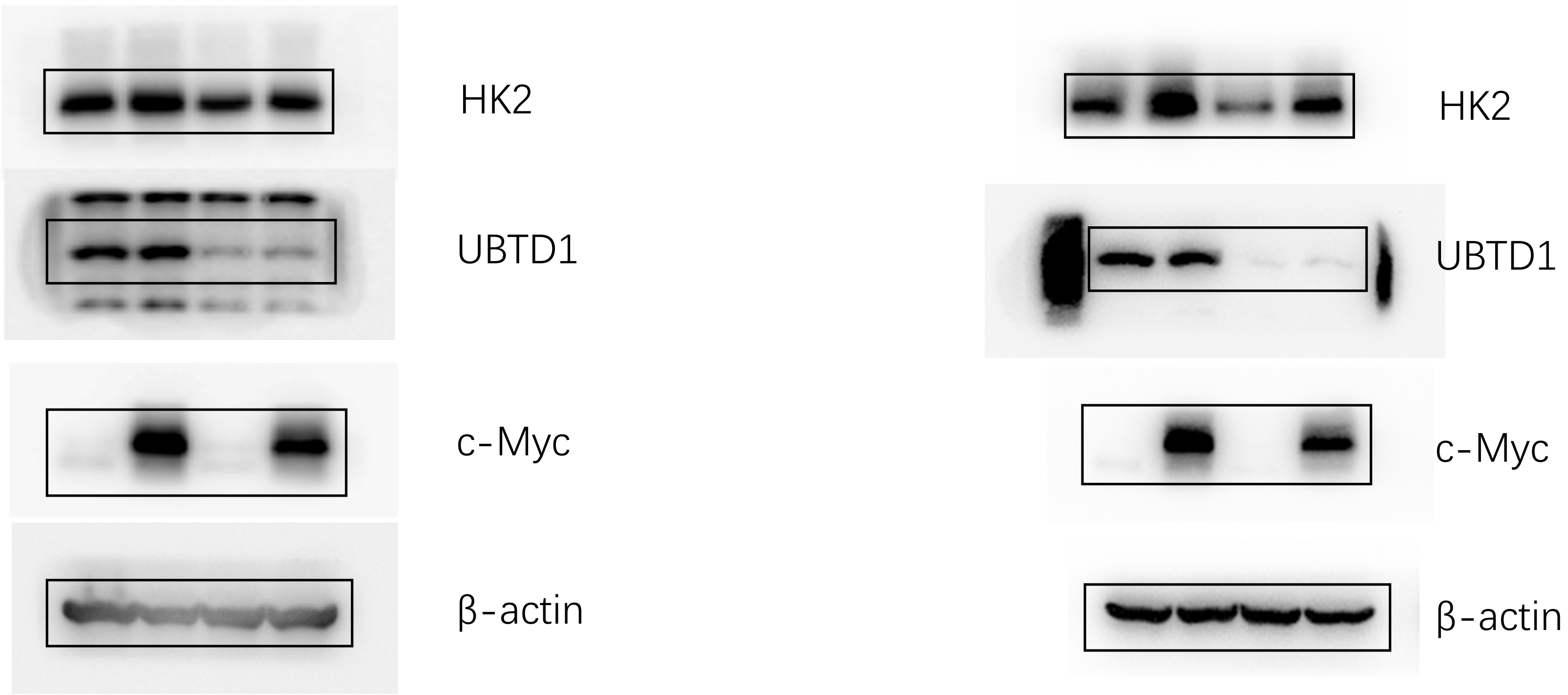

Figure 6A

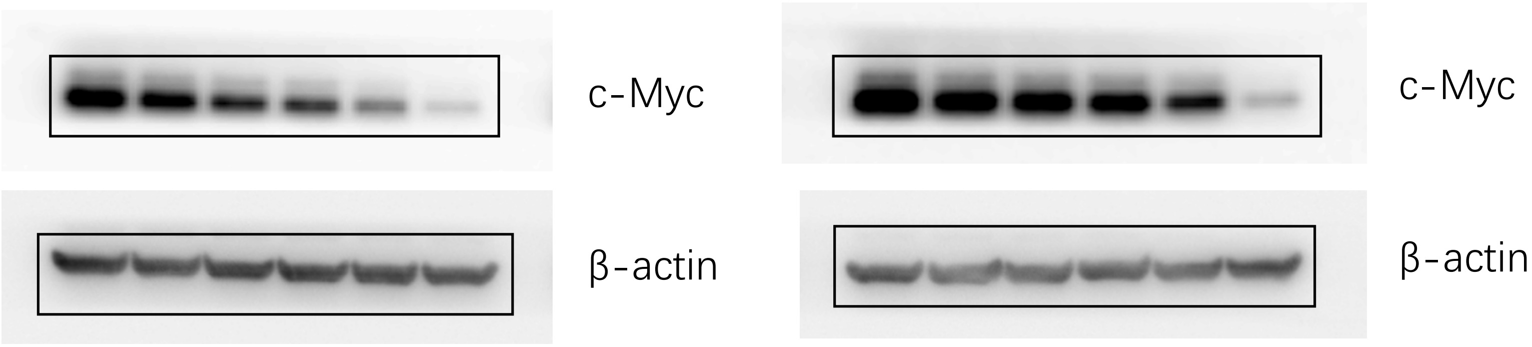

Figure 6B

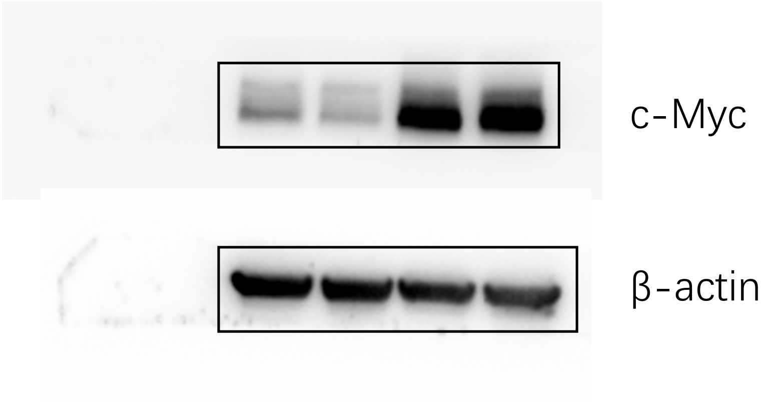

Figure 6C

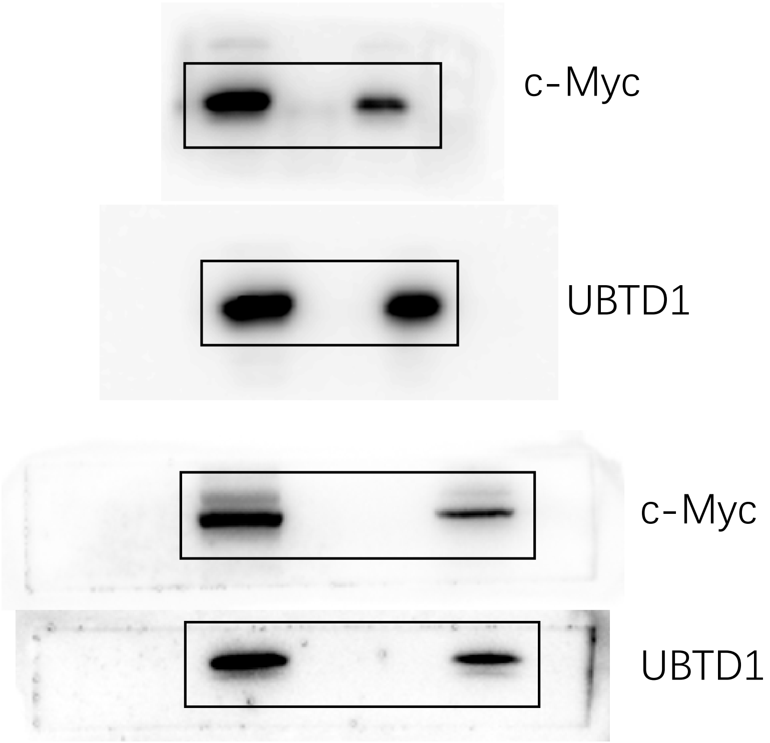

Figure 6D

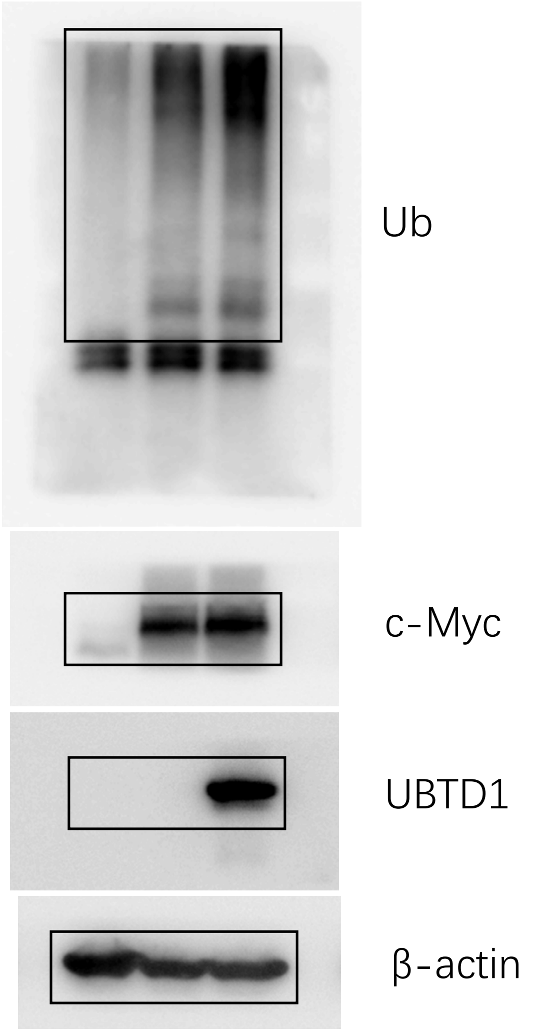

Figure 6E

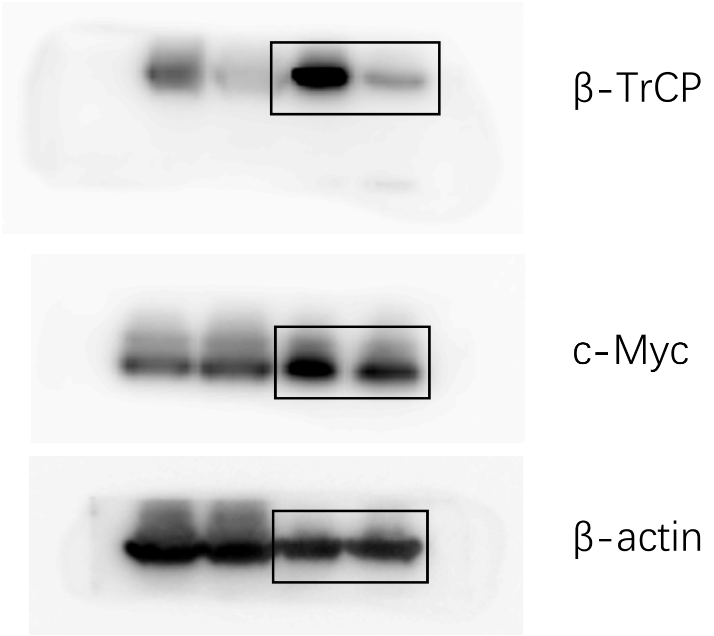

Figure 6F

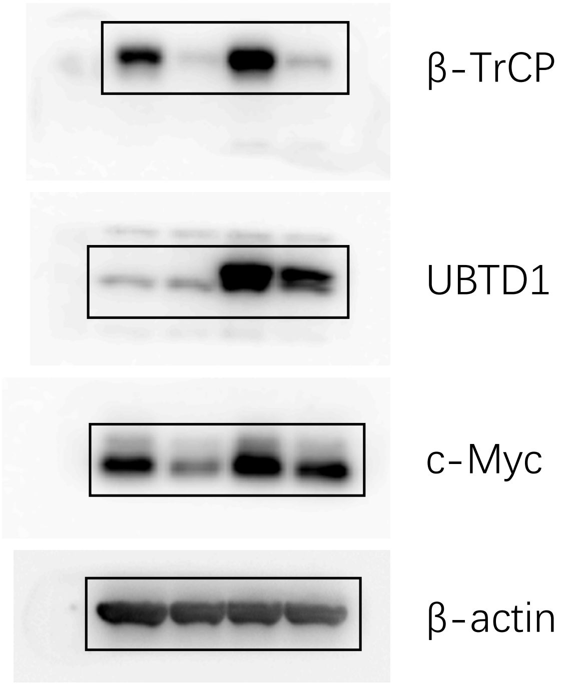

Figure 6G

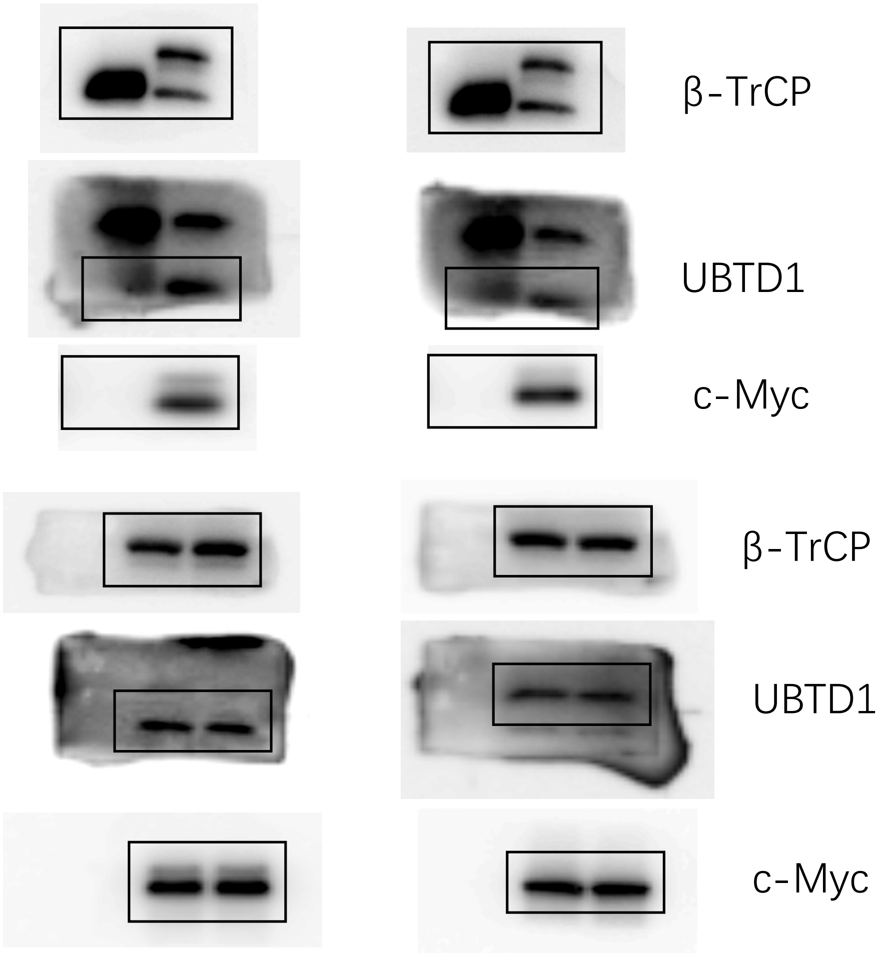

Figure 6H

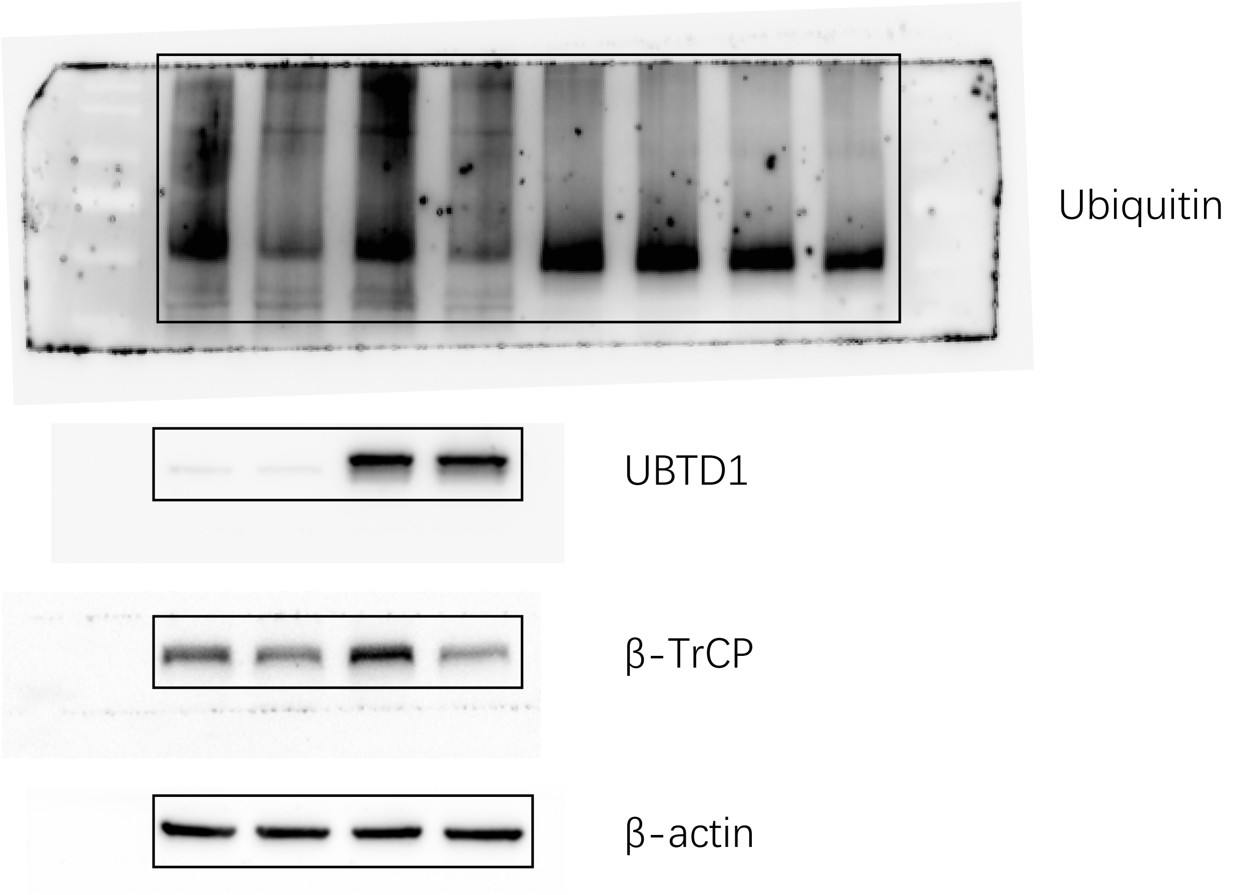

Supplementary figure 2

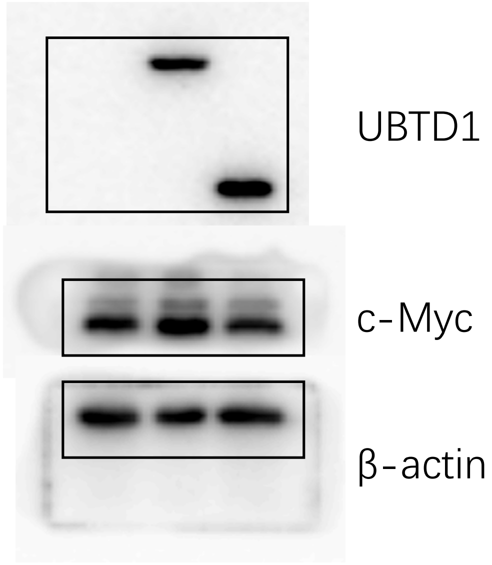

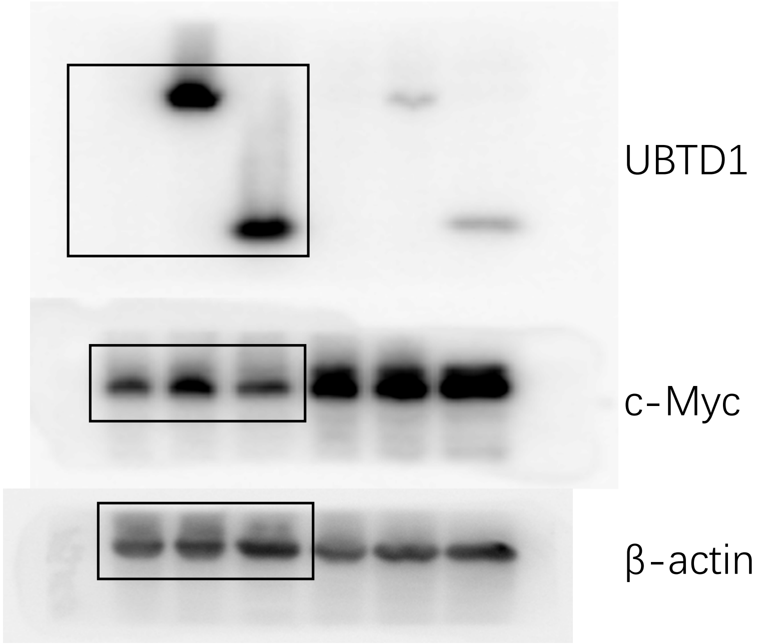

Supplementary figure 6

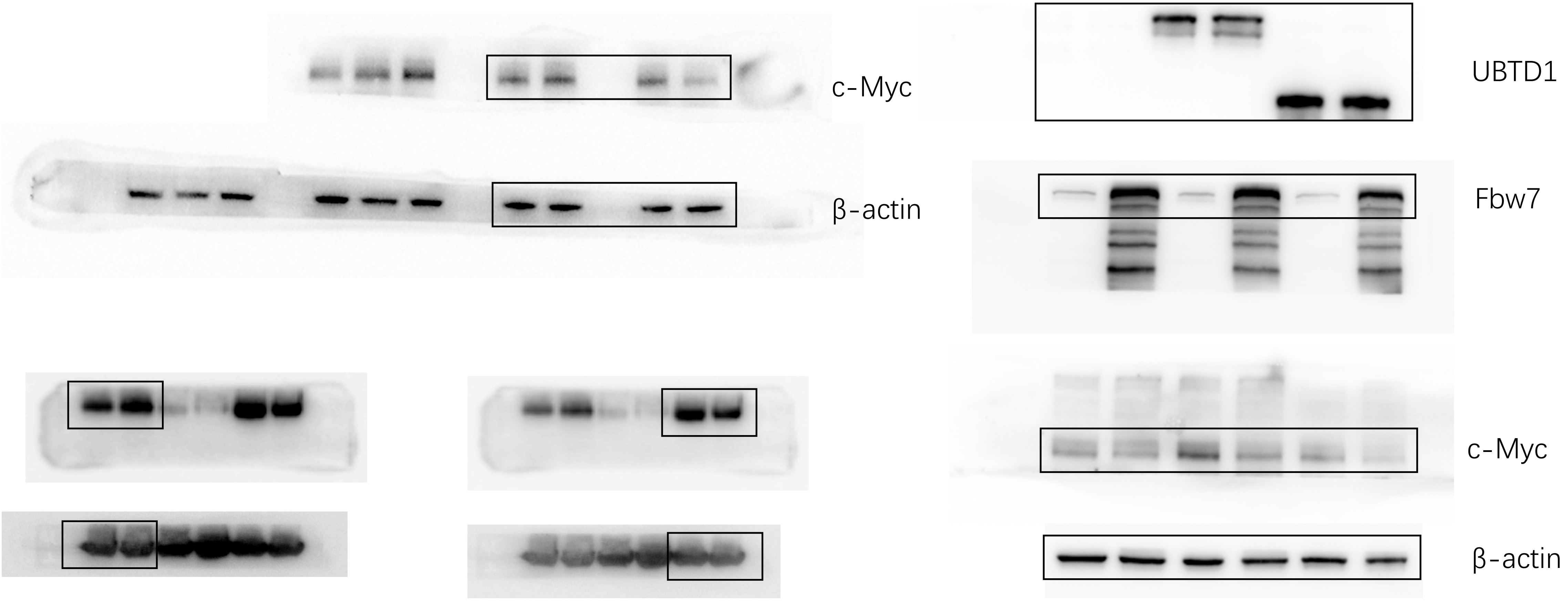

Supplement: Supplementary file 12 — uncropped original western blots [file 41419_2024_6890_MOESM12_ESM.pdf]
